# *Helicobacter pylori *interferes with an embryonic stem cell micro RNA cluster to block cell cycle progression

**DOI:** 10.1186/1758-907X-2-7

**Published:** 2011-10-25

**Authors:** Cédric Belair, Jessica Baud, Sandrine Chabas, Cynthia M Sharma, Jörg Vogel, Cathy Staedel, Fabien Darfeuille

**Affiliations:** 1 Univ. Bordeaux, ARNA Laboratory, F-33000, Bordeaux, France; 2 INSERM, U869, ARNA Laboratory, F-33000, Bordeaux, France; 3 Institute for Molecular Infection Biology, Research Centre of Infectious Diseases, University of Würzburg, Würzburg, Germany

**Keywords:** microRNAs, cell cycle, *Helicobacter pylori*, gastric cancer

## Abstract

**Background:**

MicroRNAs, post-transcriptional regulators of eukaryotic gene expression, are implicated in host defense against pathogens. Viruses and bacteria have evolved strategies that suppress microRNA functions, resulting in a sustainable infection. In this work we report that *Helicobacter pylori*, a human stomach-colonizing bacterium responsible for severe gastric inflammatory diseases and gastric cancers, downregulates an embryonic stem cell microRNA cluster in proliferating gastric epithelial cells to achieve cell cycle arrest.

**Results:**

Using a deep sequencing approach in the AGS cell line, a widely used cell culture model to recapitulate early events of *H. pylori *infection of gastric mucosa, we reveal that hsa-miR-372 is the most abundant microRNA expressed in this cell line, where, together with hsa-miR-373, it promotes cell proliferation by silencing large tumor suppressor homolog 2 (LATS2) gene expression. Shortly after *H. pylori *infection, miR-372 and miR-373 synthesis is highly inhibited, leading to the post-transcriptional release of LATS2 expression and thus, to a cell cycle arrest at the G1/S transition. This downregulation of a specific cell-cycle-regulating microRNA is dependent on the translocation of the bacterial effector CagA into the host cells, a mechanism highly associated with the development of severe atrophic gastritis and intestinal-type gastric carcinoma.

**Conclusions:**

These data constitute a novel example of host-pathogen interplay involving microRNAs, and unveil the couple LATS2/miR-372 and miR-373 as an unexpected mechanism in infection-induced cell cycle arrest in proliferating gastric cells, which may be relevant in inhibition of gastric epithelium renewal, a major host defense mechanism against bacterial infections.

## Background

MicroRNAs (miRNAs) are small endogenous non-coding RNAs that have recently emerged along with small interfering RNAs (siRNAs) as key components of the RNA silencing machinery in eukaryotes. Most of them are involved in the tight control of development and cell cycle progression [1,2], and are frequently deregulated in severe pathologies, notably in cancers [3,4]. However, the importance of RNA silencing has been primarily demonstrated in plant defense mechanisms against viruses, where it protects the host by processing long double-stranded (ds)RNAs into siRNAs which, like miRNAs, are loaded onto Argonaute-RNA induced silencing complexes (AGO-RISC) [5] to downregulate gene expression and therefore inhibit viral replication. Recently, evidence has been found to show that miRNAs are also implicated in host defense against diverse pathogens, including viruses, bacteria and parasites. For example, the human hsa-miR-32 counteracts the accumulation of primate foamy virus type 1 (PFV-1) in human cells, targeting directly the PFV-1 genome and inhibiting its translation [6]. Similarly, the *Arabidopsis thaliana *miR-393, a pathogen-associated molecule pattern (PAMP)-responsive miRNA, contributes to the resistance of the plant against a virulent *Pseudomonas syringae (P. syringae) *bacterial strain [7]. In response, pathogens have evolved strategies to counteract RNA silencing. A number of pathogen-derived RNA silencing suppressors have been described in eukaryotes, such as the p19 protein of the tomato bushy stunt virus, which selectively sequesters short double-stranded RNA (dsRNA) [8], the Tas protein of PFV-1, which blocks the miRNA-directed silencing [6] or the AvrPto, AvrPtoB and HopT1 proteins of *P. syringae*, which suppress miR-393a/b biogenesis and activity [9].

In mammals, the miRNA pathway is a key actor in innate immunity, and viruses are able to exploit the non-antigenic potential of miRNAs to favor their replication [10]. Two miRNAs, miR-146a and miR-155, are major regulators of innate immune responses in monocytes/macrophages [11-13]. Both are strongly induced through Toll-like receptor (TLR) engagement after PAMP recognition and nuclear factor (NF)κB activation. In turn, they target the TLR signaling cascades and thus, moderate the life-threatening overproduction of inflammatory cytokines through a negative feedback loop. Recently, the *let-7 *family has been identified as a new actor of the innate response [14]. Indeed, during *Salmonella *infection of murine macrophages, TLR4 signaling triggered by bacterial lipopolysaccharide (LPS) leads to the repression of *let-7 *family and results in the expression of its targets, the proinflammatory interleukin (IL)-6 and the anti-inflammatory IL-10, thus modulating tightly the immune response [14,15].

However, the epithelial barrier constitutes the first line of defense against pathogens. In contrast to results obtained in immune cells, miR-155 and miR-146 were not found upregulated in mouse lungs, among the numbers of miRNAs rapidly induced after bacterial LPS exposure, suggesting that epithelial cells may express a different set of miRNAs than immune cells in response to pathogens [16]. The importance of miRNAs in epithelial defense against pathogens was highlighted in biliary epithelial cells infected with the protozoan parasite *Cryptosporidium parvum*, an infection model in which let-7i and miR-513 contribute to the epithelial immune response. Indeed, upon *C. parvum *infection, these miRNAs are downregulated, in a MyD88/NFκB-dependent manner for let-7i, leading to the upregulation of TLR4 and B7-H1 expression, respectively [17-19].

*Helicobacter pylori *is a Gram-negative microaerophilic bacterium which chronically infects the gastric mucosa of about half of the world population, and constitutes the primary etiological cause of gastritis, peptic ulcer and gastric cancers [20]. The most virulent strains, associated with a higher risk of gastric adenocarcinomas [21], harbor in their genome a cluster of 31 genes called the *cag *pathogenicity island (*cag*PAI), which encode a type IV secretion system (T4SS) and the CagA toxin. This T4SS interacts with host cell surface and injects the CagA protein into the cell cytoplasm [22]. The translocated CagA interferes then with intracellular signaling pathways, which subsequently alters cell-to-cell adhesion, cell polarity and proliferation [23-25]. A functional T4SS is also essential for the induction of innate immune response, characterized by NFκB activation and IL-8 production in gastric epithelial cells [26].

The extent to which miRNAs contribute to the gastric epithelial cell response to *H. pylori *infection has been little explored to date [27]. So far, only two miRNAs, miR-21 and miR-155, have been reported upregulated in both *H. pylori*-infected human gastric mucosa and i*n vitro *cell culture [28,29]. As the gastric epithelial cells are the first line of defense against *H. pylori*, triggering the immune response, it is relevant to further assess how miRNA are modulated upon *H. pylori *infection in these cells. With this aim, we used a common gastric epithelial cell model, the human AGS cell line [30]. First, we employed a deep-sequencing approach to identify the AGS miRNAs profile in basal and infection conditions. Surprisingly, the most abundant one, miR-372, belongs to a miRNA cluster, miR-371-372-373, specifically expressed in embryonic stem cells. Moreover, this cluster is repressed upon *H. pylori *infection in a CagA-dependent manner. The repression of miR-372 and miR-373 is associated with the upregulation of their target, the cell cycle regulator large tumor suppressor homolog 2 (LATS2), leading to the inhibition of cell cycle progression in *H. pylori-*infected cells.

## Results

### AGS cell line expresses a particular set of miRNAs

**Figure 1:**
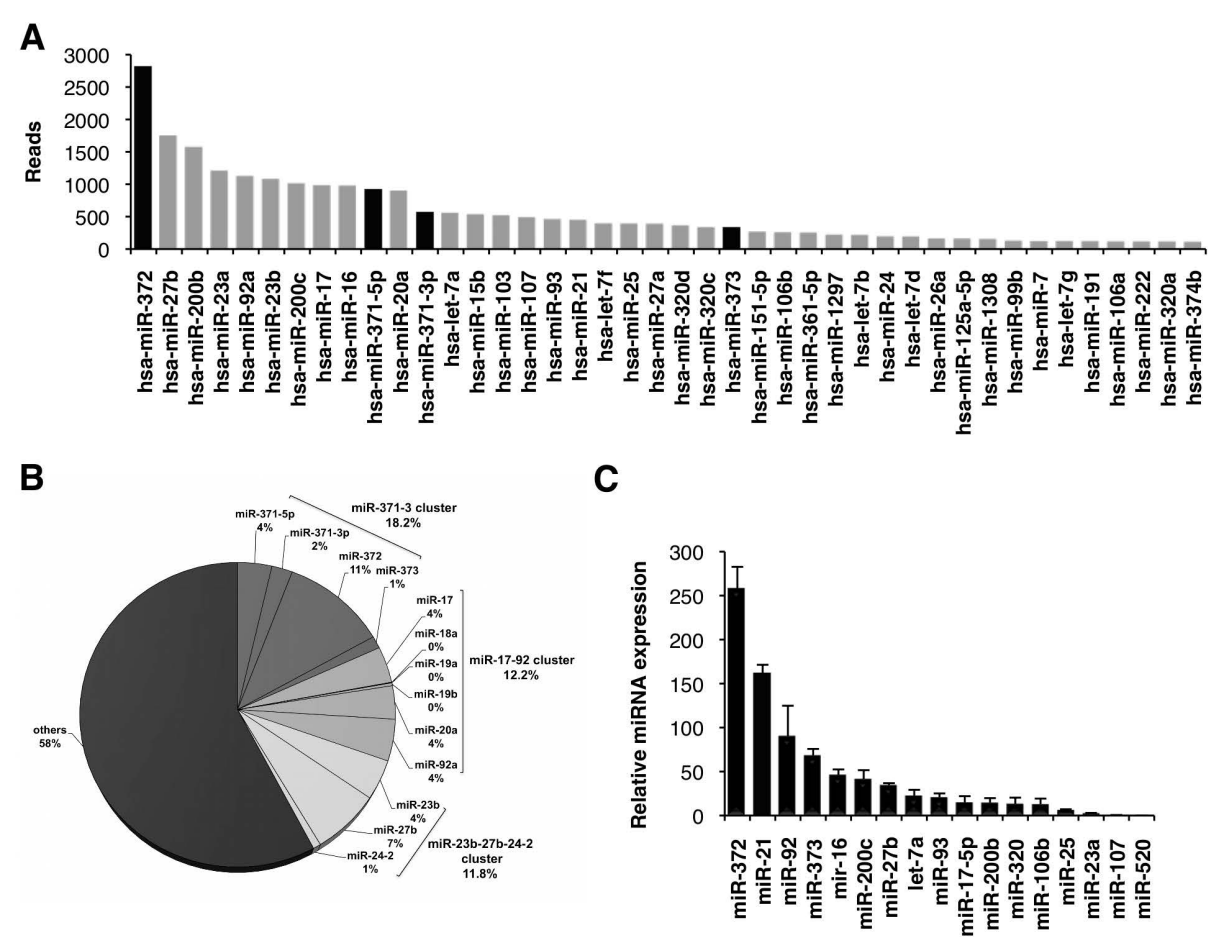
**Micro RNA (miRNA) expression profile in AGS cells**. **(A) **miRNA content of AGS cells was determined by high-throughput '454' pyrosequencing [see Additional File 1, Table S1]. Bars indicate the number of reads for each miRNA out of 25,348 miRNA matching sequences. Only the 42 most abundant miRNAs are plotted in order of decreasing number of reads. Black bars indicate mature miRNAs belonging to the miR-371-372-373 cluster. **(B) **Pie chart represents the percentage and clustering of the most highly expressed miRNAs in AGS cells. Percentage represents the number of reads of each miRNA relative to the total number of reads. **(C) **Endogenous expression of some mature miRNAs in AGS cells was measured by quantitative real time PCR (RT-qPCR). Bars indicate the relative expression of each miRNA normalized to U6 small nuclear RNA (snRNA) (RNU6B). Data are mean ± SD of three independent experiments.

The AGS cell line is widely used in *in vitro *experiments to recapitulate early events of *H. pylori *infection occurring within actively replicating gastric mucosa [31,32]. To address the complete miRNA profile of the AGS cell line, we employed a high-throughput pyrosequencing approach. A cDNA library of growing AGS cells was generated and submitted to '454' technology. A total of 44,012 sequences were analyzed, resulting in 25,348 (57.6%) sequences matching with mature human miRNA sequences of the miRBase 14.0 [see Additional file 1, Table S1]. The other sequences corresponded to various RNA degradation products or unidentified sequences. Mature miRNAs identified with this method can be classified in function of their respective number of reads (Figure 1A), their genomic organization in clusters (Figure 1B) or their function previously assigned to them in other studies [see Additional file 1, Table S2]. We decided to restrict our analysis to the 38 most abundant miRNAs (>100 reads, Figure 1A). Consistent with their respective genomic annotations, many of the mature miRNAs identified were organized in clusters. Thus, the miRNA signature of AGS cells can be summarized into three major clusters: the miR-371-372-373 cluster, the miR-17-92 cluster and the miR-23b-27b-24-2 cluster, which represent 18.6%, 12.4% and 12.1% of total number of reads, respectively (Figure 1B). Members of the miR-200 and *let-7 *families were also highly represented: miR-200b, miR-200c and let-7a being the most expressed members of these families in AGS cells [see Additional file 1, Table S1].

The expression of some of these miRNAs was confirmed by quantitative real time PCR (RT-qPCR) (Figure 1C) and northern blot (data not shown). As expected, all tested miRNAs present in our miRNA sequencing library were also detected by these techniques. However, two miRNAs, miR-155 and miR-146a, reported to be present in AGS cells [33] and absent from our library were weakly detected by RT-qPCR (close to the detection threshold; data not shown), suggesting that our library might lack some miRNAs faintly expressed in this cell line.

### miR-372 and miR-373 regulate LATS2 expression in AGS cells

The peculiarity of the AGS miRNA repertoire, which besides containing ubiquitous miRNAs such as miR-16, miR-21, miR-17 and miR-92, resides in the outstanding abundance of miR-372, which was present in the range of 10^6 ^copies/ng total RNA or 10^4 ^copies per cell (see Methods). Intriguingly, miR-372 is an embryonic stem-cell-specific miRNA [34], also found highly expressed in placenta [35]. It belongs to a cluster located on the chromosomic region 19q13.42, which comprises four miRNAs, all of them highly expressed in AGS cells: miR-371-5p, miR-371-3p, miR-372 and miR-373 (Figure 1A, black bars). miR-372 or miR-373 expression is considered as a rare event in tumors, and has only been found in testicular germ cell tumors [36], esophageal tumors [37] and thyroid adenomas [38].

**Figure 2:**
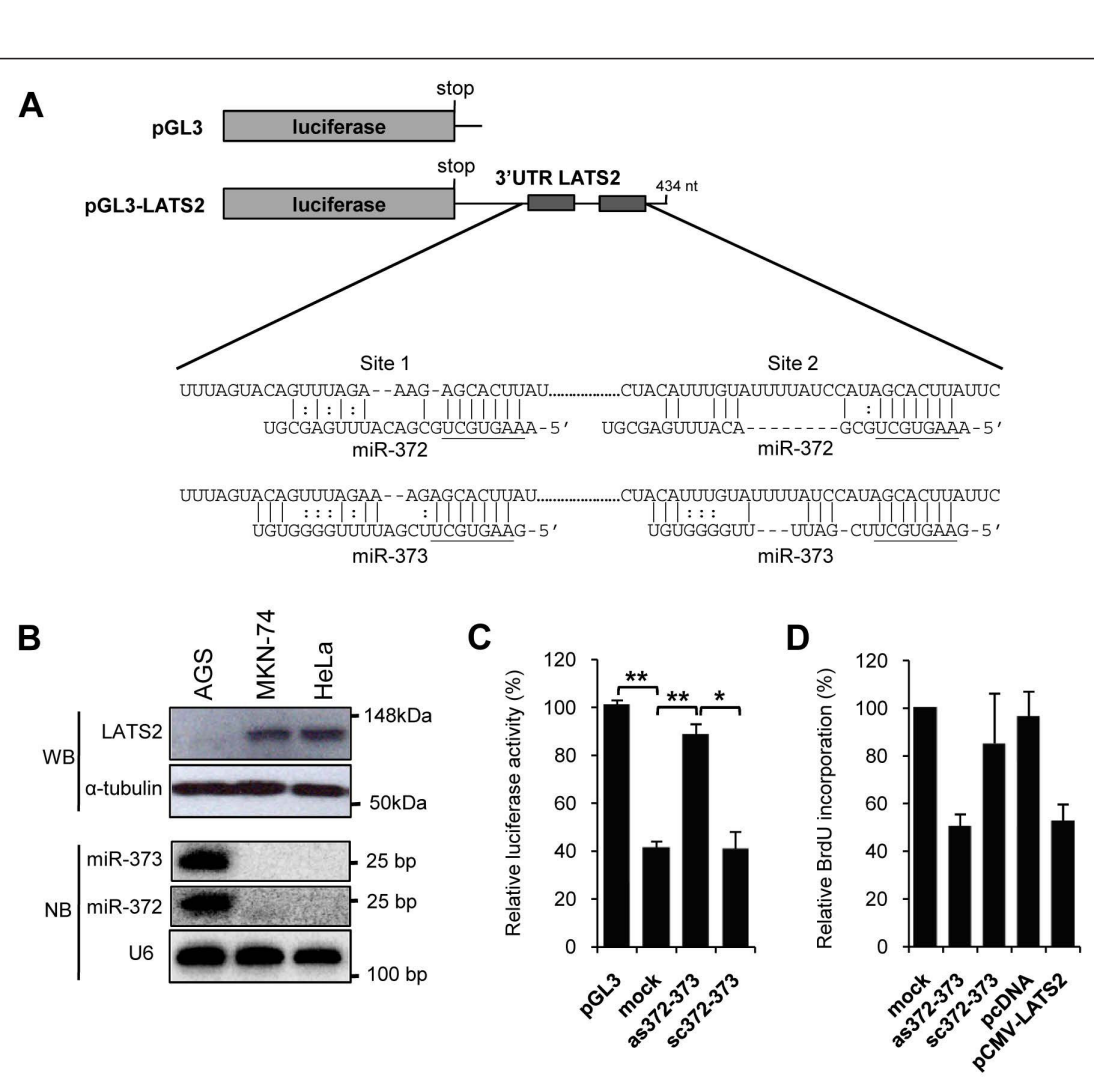
**miR-372 and miR-373-mediated regulation of large tumor suppressor homolog 2 (LATS2) translation and AGS cell proliferation**. **(A) **Schematic representation of the pGL3 vector (control) and the pGL3-LATS2 sensor containing the 3' untranslated region (UTR) of the LATS2 human gene, which harbors two predicted miR-372 and miR-373 target sequences [36]. Also shown is the predicted base pairing formed between miR-372 or miR-373 with each of the LATS2 3'UTR binding sites as predicted by http://pictar.mdc-berlin.de. Seed regions of miR-372 and miR-373 are underlined. **(B) **LATS2 levels and mature miR-372 and miR-373 expression in AGS, MKN-74 and HeLa cells were determined by immunoblot and northern blot, respectively. α-Tubulin and U6 small nuclear RNA (snRNA) were used as loading controls. **(C) **The pGL3 and pGL3-LATS2 vectors were transfected into either parental AGS (mock), or cells treated with 100 nM of as372-373 or the sc372-373 control oligonucleotides. Transfection efficiency was assessed with the pRL-SV40 vector. Luciferase activities were measured 48 h post transfection. Bars indicate the relative firefly luciferase activity normalized to the *Renilla *activity and compared to pGL3. Data are mean ± SD from three independent experiments. *P < 0.05; **P < 0.01. **(D) **The DNA synthesis rate was measured by 5-bromo-2'-deoxyuridine (BrdU) incorporation (10 nM for 1 h) in untreated (mock) AGS cells or cells treated for 48 h with 100 nM as372-373 or sc372-373 or transfected with the pCMVmyc-LATS2 vector. Bars represent the relative BrdU incorporation compared to that of untreated cells. Data are mean ± SD of three independent experiments. **P < 0.01.

In testicular and esophageal tumors, in which they are abundant, miR-372 and miR-373 have been reported to act as oncogenes repressing LATS2, a serine-threonine kinase involved in cell cycle regulation [36,37]. On performing immunoblot analysis of LATS2, we did not detect LATS2 in AGS cells, in contrast to the other gastric cell line MKN-74 and the cervix cancer cell line HeLa (Figure 2B, upper panel). It appears that the LATS2 level was inversely correlated to the expression of miR-372 and miR-373 in these cell lines (Figure 2B, lower panel). These results suggest that LATS2 could be repressed at the post-transcriptional level by miR-372 and miR-373 in AGS cells. To validate this hypothesis, we transfected into AGS cells the luciferase sensor pGL3-LATS2, containing the 3' untranslated region (UTR) of LATS2, which harbors two miR-372 and miR-373 pairing sites [36] downstream to the firefly luciferase coding sequence (Figure 2A). As expected from cells expressing high miR-372 and miR-373 levels, we observed a 60% inhibition of luciferase expression, as compared to the control pGL3 reporter (Figure 2C). No such inhibition was observed when the pGL3-LATS2 vector was transfected in MKN-74 cells, which express very low miR-372 and miR-373 levels [see Additional File 2, Figure S1]. This repression was dependent on the two miR-372 and miR-373 binding sites located in the LATS2 3'UTR, as no significant inhibition was observed in AGS cells transfected with the pGL3-LATS2mut vector mutated for these sites [see Additional File 2, Figure S1]. To confirm the role of miR-372 and miR-373 in this repression, we designed antisense oligonucleotides (as372, as373) and their scrambled controls (sc372, sc373) in order to block these miRNAs. The levels of detectable miR-372 or miR-373 were each specifically decreased by 90% by as372/as373, as verified by RT-qPCR and northern blot analysis [see Additional File 3, Figure S2A,B]. This decrease leads to an accumulation of LATS2 protein in as372-373-treated cells, compared to sc372-373-treated cells [see Additional File 3, Figure S2D]. We transfected either pGL3-LATS2, or the control pGL3 vector, together with as372-373 or the control sc372-373, and analyzed the luciferase activity. In as372-373 treated cells, the firefly luciferase expression was significantly derepressed and almost reached the levels of that of the control vector (Figure 2C), contrarily to the cells treated with sc372-373, which did not affect the basal miR-372 or miR-373 levels. Altogether, these results indicate that LATS2 is indeed a direct target of miR-372 and miR-373 in AGS cells.

Inhibition of LATS2 by miR-372 and miR-373 confers a growth advantage to cells [36,37]. To confirm that LATS2 is a functional target of miR-372 and miR-373 in AGS cells, we analyzed cell cycle progression of either as372-373-treated or LATS2-transfected cells using the 5-bromo-2'-deoxyuridine (BrdU) incorporation method, which measures the rate of DNA synthesis occurring in the S phase of the cell cycle. The as372-373-treated AGS cells presented a significantly delay of about 50% in S phase progression compared to sc372-373 treated cells (Figure 2D). Similarly, overexpression of LATS2 [see Additional File 3, Figure S2D] by transfecting the pCMVmyc-LATS2 vector, which allows the expression of the human LATS2 cDNA, resulted in a similar inhibition of BrdU incorporation see Additional File 3, Figure S2E]. The delays in cell cycle progression exhibited by either as372-373-transfected or LATS2-transfected cells resulted in decreased cell proliferation rates of AGS cells [see Additional File 3, Figure S2C,E], whereas the growth of MKN-74 cells, which expressed LATS2 in basal conditions, was not inhibited by the ectopic expression of LATS2 [see Additional File 3, Figure S2E]. Altogether, these results indicate that LATS2 is a functional target of miR-372 and miR-373 in AGS cells and that the high expression of miR-372 and miR-373 confers to them a growth advantage through the inhibition of LATS2 expression.

### The miR-371-372-373 cluster is downregulated in response to *H. pylori *infection

**Figure 3:**
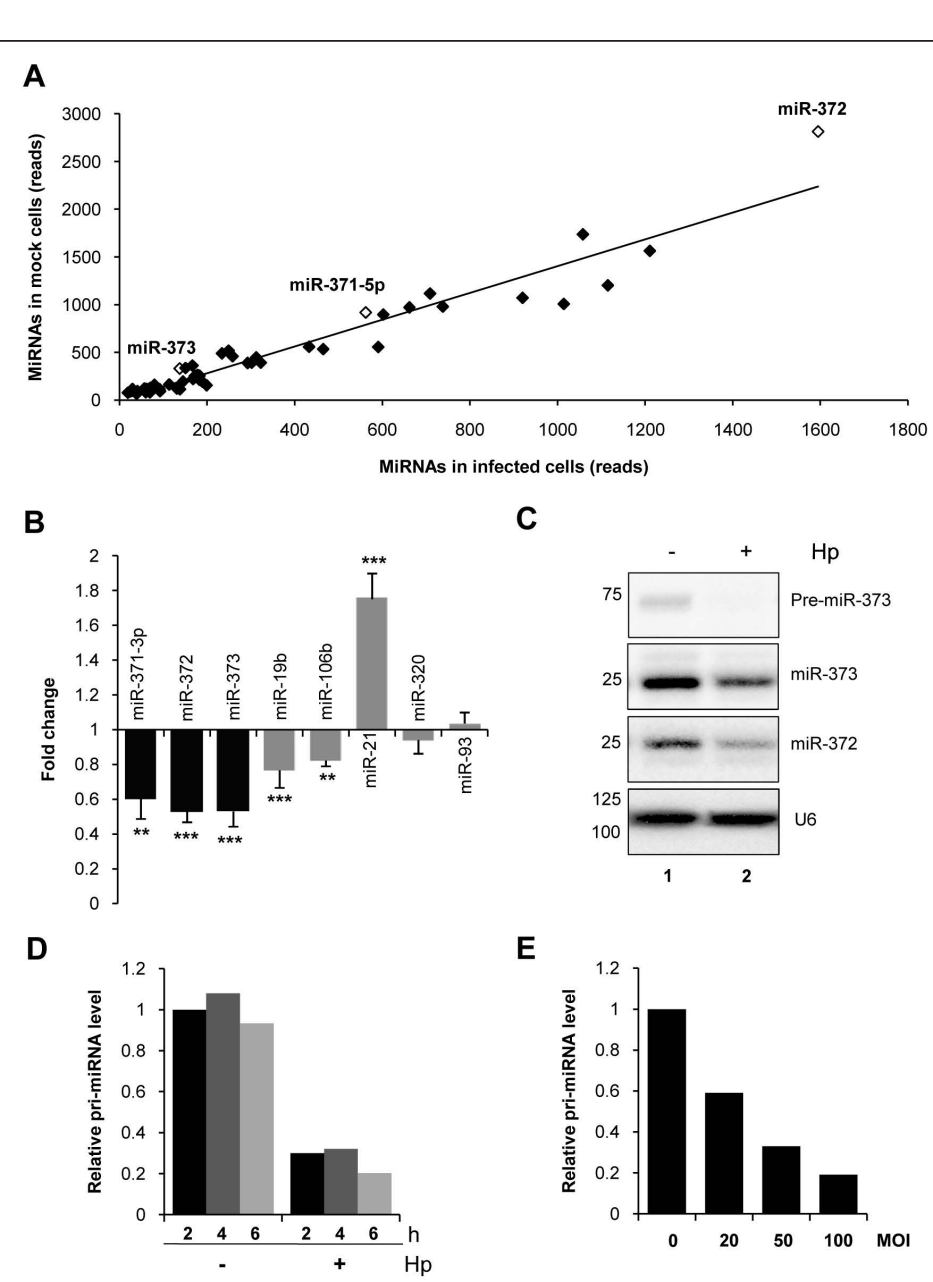
***Helicobacter pylori*-induced downregulation of mature miR-371, miR-372 and miR-373 levels in AGS cells**. **(A) **Micro RNAs (miRNAs) content of AGS cells cocultured with *H. pylori *was determined by high-throughput '454' pyrosequencing. The graph represents the miRNAs content in reads of uninfected AGS cells (y axis) versus infected cells (x axis). miR-372, miR-373 and miR-371-5p are shown (empty plots). The trend line is represented, R^2 ^= 0.9556 [see Additional File 1, Table S1 for total number of reads of each miRNA and Fisher's exact test *P *values]. **(B) **Endogenous expression of mature miR-371-3p, miR-372 and miR-373 (black bars), or miR-19b, miR-106b, miR-21 and miR-93 (grey bars), was determined by quantitative real time PCR (RT-qPCR) in AGS cells 24 h post infection at multiplicity of infection (MOI) 100. Bars indicate the relative miRNA expression normalized to SNORD49A (RNU49) and compared to uninfected AGS cells. Data represent mean ± SD of three independent experiments. **P < 0.01; ***P < 0.001. **(C) **Northern blot analysis of mature miR-372, miR-373 and pre-miR-373 expression in uninfected (lane 2) or *H. pylori*-infected AGS cells (lane 3) in the same conditions as in (A). lane 1: M, size marker (nucleotides). A representative blot is shown. **(D,E) **Expression of the primary miR-371-372-373 cluster transcript after different time of infection (MOI 100) (D), and different MOI (6 h post infection) (E). Bars indicate the relative primary polycistronic miRNA transcript (pri-miRNA) expression determined by RT-qPCR, normalized to the ribosomal protein P0 mRNA and compared to uninfected AGS cells. A representative experiment is shown.

To analyze miRNA changes in AGS cells upon *H. pylori *infection, a cDNA library of AGS cells cocultured with the bacteria for 5 h was generated and also submitted to '454' pyrosequencing. In all, 43,863 sequences were analyzed, resulting in 17,778 (40.6%) sequences matching with mature human miRNA sequences of the miRBase 14.0 [see Additional File 1, Table S1]. The pyrosequencing data were analyzed by a two-dimensional scatter plot, which compares the number of reads for a same miRNA from uninfected and infected AGS cells (Figure 3A). The majority of miRNAs appear along the trend line, indicating that they were unaffected by the infection. The miRNAs affected by *H. pylori *appear either above (downregulated) or below (upregulated) the trend line. Interestingly, miR-372, which is the most abundant miRNA expressed in AGS cells, was also the most significantly repressed upon infection (Figure 3A, Fisher's exact test, *P *value = 5.1 × 10^-13^). Other members of the miR-371-372-373 cluster, miR-373 and miR-371-5p, also appeared significantly downregulated upon infection (Figure 3A, *P *values = 8.3 × 10^-8 ^and 0.009, respectively). These results suggest that this cluster is highly repressed upon infection and prompted us to further analyze its regulation upon *H. pylori *infection. With this aim, AGS cells were cultured in the presence of the *H. pylori *26695 strain at a multiplicity of infection (MOI) of 100 and the miR-371-372-373 cluster expression was analyzed 24 h later. Mature miR-371-3p, miR-372 and miR-373 were all significantly downregulated (up to 50%) upon infection, as shown by RT-qPCR and northern blot analysis (Figure 3B,C, respectively). This repression was not the consequence of a global effect on the miRNA pathway, as some other miRNAs were differently modulated upon infection. For example, miR-106b and miR-19b were slightly repressed while miR-21 was upregulated upon infection, in concordance with prior observations [29]. We also found miRNA levels unchanged upon infection, such as miR-93 and miR-320 (Figure 3B). Downregulation of miR-372 and miR-373 upon infection was also shown by northern blot analysis (Figure 3C), which in addition revealed that miRNA precursors (pre-miRNA) were even more strongly repressed than the mature forms (Figure 3C, upper part of the blot for miR-373). These findings suggest that the regulation took place at an initial step of miR-371-372-373 biogenesis. We confirmed this hypothesis by RT-qPCR on the primary polycistronic miRNA transcript (pri-miRNA) of the cluster (Figure 3D,E). Indeed, the pri-miRNA was rapidly downregulated upon *H. pylori *infection, as soon as 2 h post-infection at MOI 100 (Figure 3D), and in a MOI-dependent manner (Figure 3E). Altogether, these results show that the miR-371-372-373 cluster was strongly decreased at the pri-miRNA level shortly upon *H. pylori *infection.

### miR-371-372-373 cluster is repressed in a CagA-dependent manner

**Figure 4:**
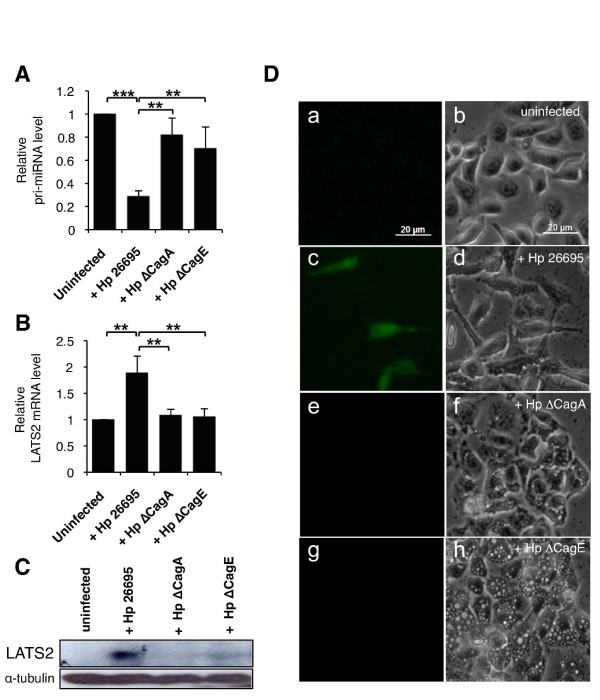
**CagA-dependent repression of primary (pri-)miR-371-372-373 and upregulation of large tumor suppressor homolog 2 (LATS2) expression **. **(A,B) **Expression of the primary miR-371-372-373 cluster transcript (A) or LATS2 mRNA (B) was determined by quantitative real time PCR (RT-qPCR) in uninfected AGS cells or cells infected for 24 h at multiplicity of infection (MOI) 100 with either *Helicobacter pylori *26695 or its ΔCagA or ΔCagE isogenic mutants. Bars indicate the relative pri-micro RNA (miRNA) or LATS2 mRNA expression, normalized to the ribosomal protein P0 mRNA and compared to uninfected AGS cells. Data are mean ± SD of three independent experiments. **P < 0.01; ***P < 0.001. **(C) **LATS2 immunoblot of AGS cells infected or not with *H. pylori *or its isogenic mutants as described above. α-Tubulin was used as a loading control. **(D) **Enhanced green fluorescent protein (EGFP) fluorescence of LATS2 reporter cells infected or not with wild-type *H. pylori*, or the ΔCagA or ΔCagE isogenic mutants. Cells were infected as described above and observed 24 h later on an inverted microscope (Zeiss) in phase contrast (right panels) or epifluorescence (left panels).

To assess whether the downregulation of the miR-371-372-373 cluster was related to a functional *H. pylori cag*PAI, we compared the effect of wild-type bacteria to those of isogenic mutants deleted either for the *cagA *(ΔCagA) or for the T4SS constituent *cagE *(ΔCagE) genes. As previously described [26,39], these mutants like wild-type *H. pylori *induced vacuolization of AGS cells, an effect due to the vacuolating toxin VacA, but failed to provoke cell elongation and scattering, the so-called 'hummingbird' phenotype, characteristic of AGS cells infected with wild-type *H. pylori *[see Additional File 4, Figure S3A]. Moreover, these mutants were impaired in their proinflammatory effects on AGS cells, as measured by NFκB activation and IL-8 secretion [see Additional File 4, Figure S3B,C]. Interestingly, both mutants have a very weak effect on the miR-371-372-373 cluster (Figure 4A). Indeed, when AGS cells were cocultivated with the ΔCagA or ΔCagE mutants, which are, respectively, unable to produce the CagA virulence factor or to inject it into the host cell cytoplasm, the pri-miRNA was not repressed, compared to cells infected with wild-type *H. pylori*. These data show that the downregulation of the miR-371-372-373 cluster upon *H. pylori *infection is mediated by the translocation of CagA into AGS cells.

To evaluate the effects of the downregulation of miR-372 and miR-373, we analyzed the regulation of their common target, LATS2, in AGS cells upon *H. pylori *infection. We observed that LATS2 mRNA (Figure 4B) and protein levels (Figure 4C) were inversely correlated to those of miR-372 and miR-373 upon *H. pylori *infection. Indeed, LATS2 protein was drastically upregulated in infected AGS cells and, likewise, its mRNA level was also raised up to twofold upon infection. Moreover, similarly to the pri-miR-371-372-373 repression, LATS2 accumulation was dependent on CagA, as no changes in LATS2 expression were observed either at the mRNA and protein levels in AGS cells infected with the ΔCagA or ΔCagE mutants, conversely to wild-type *H. pylori *(Figure 4B,C).

To confirm that LATS2 synthesis was derepressed at the post-transcriptional level after miR-372 and miR-373 downregulation in AGS cells infected with wild-type *H. pylori*, we generated a stable LATS2 reporter AGS cell line expressing a fluorescent sensor containing the 3'UTR of LATS2 downstream of the enhanced green fluorescent protein (EGFP). This reporter system senses changes in the miR-372 and miR-373 levels and allows a direct observation of LATS2 post-transcriptional regulation in living cells. Indeed, as372-373-treated cells, in which miR-372 and miR-373 levels were decreased, turn fluorescent, whereas sc372-373 did not [see Additional File 5, Figure S4]. As depicted in Figure 4D (panel c), green fluorescence appeared only upon infection with wild-type *H. pylori*, mainly in cells exhibiting the hummingbird phenotype (Figure 4D, panels c and d), whereas uninfected cells (Figure 4D, panels a and b) or cells infected with the ΔCagA or ΔCagE mutant strains (Figure 4D, panels e and g) remained non-fluorescent. We also used this reporter cell line to assess whether other strains, naturally expressing CagA (type I) or not (type II), were able to induce LATS2 expression. Indeed, the P12 type I strain was able to switch on the fluorescence of the reporter cells as the 26695 strain did, whereas type II strains, such as SS1 and X47-2AL, did not [see Additional File 6, Figure S5B]. We were also able to correlate this post-transcriptional activation with a concomitant downregulation of the pri-miR371-372-373 transcript level [see Additional File 6, Figure S5A]. These data confirm that the CagA-dependent accumulation of LATS2 protein involves a post-transcriptional release of its expression mediated by the downregulation of miR-372 and miR-373.

### miR-372 and miR-373 downregulation is involved in *H. pylori*-induced cell cycle arrest in G1 phase

**Figure 5:**
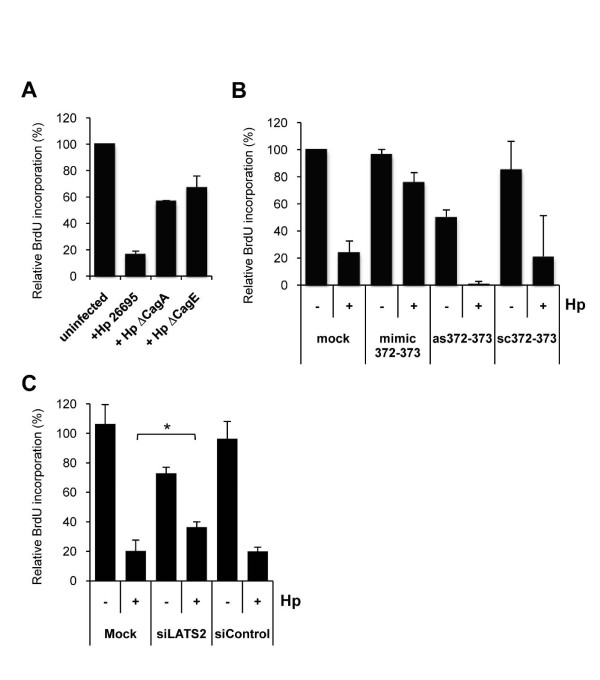
**Role of miR-372 and miR-373 in *Helicobacter pylori*-induced cell cycle arrest in AGS cells**. **(A) **DNA synthesis rate of uninfected AGS cells or cells infected for 24 h with wild-type *H. pylori *or its isogenic mutants ΔCagA and ΔCagE. **(B,C) **DNA synthesis rate of parental AGS cells (mock), or treated cells infected with or without wild-type *H. pylori *for 24 h. Previously, cells were transfected either with 100 nM of a mixture (1:1) of anti-miR-372 and miR-373 antisense oligonucleotides (as372-373), 25 nM miR-372 and miR-373 double-stranded RNA mimics or 100 nM scrambled oligonucleotides (sc372-373) (B), or treated with 20 nM of anti- large tumor suppressor homolog 2 (LATS2) siRNA or control siRNA (C). DNA synthesis level was measured by 5-bromo-2'-deoxyuridine (BrdU) pulse labeling. Bars represent the relative BrdU incorporation level compared to that of uninfected (A) or uninfected parental (B,C) AGS cells. Data are mean ± SD of two independent experiments. *P < 0.05.

To assess whether LATS2 upregulation was functionally relevant, we analyzed cell cycle progression of AGS cells upon infection. In agreement with previous observations [31], AGS cells infected for 24 h with wild-type *H. pylori *presented a noticeable alteration of the cell cycle, characterized by an accumulation of cells in the G1 phase, in the detriment of cells in S and G2/M phases [see Additional File 7, Figure S6]. Accordingly, measuring BrdU incorporation 24 h post infection, we observed an 80% reduction of AGS cells in S phase, which reflects a cell cycle arrest at the G1/S transition (Figure 5A). As already shown by Murata-Kamiya and colleagues [24], this reduction was dependent on CagA, as cells infected with the ΔCagA or ΔCagE mutants did not show such inhibition of cell cycle progression (Figure 5A [see Additional File 7, Figure S6]). To ascertain that the inhibition of G1/S transition was due to the downregulation of miR-372 and miR-373, we treated AGS cells with synthetic miR-372 and miR-373 prior to infection, in order to dampen their downregulation upon infection. In this case, the proportion of infected cells in S phase remained as high as in untreated and uninfected cells (Figure 5B), indicating that wild-type *H. pylori *became unable to arrest AGS cell cycle progression. Conversely, blocking miR-372 and miR-373 with as372-373 prior to infection facilitated the infection-induced inhibition of DNA synthesis, which then reached its maximum (Figure 5B). In addition, silencing LATS2 with siRNA against LATS2 mRNA reduced the efficiency of *H. pylori *to block AGS cell cycle (Figure 5C), confirming the LATS2 involvement in the inhibition of cell cycle progression in infected AGS cells.

The effects of these experimental manipulations of miR-372 and miR-373 and their common target LATS2 on the efficiency by which wild-type *H. pylori *blocks AGS cell cycle demonstrate that the regulation of these miRNAs contribute to the arrest at the G1/S transition in infected AGS cells. Altogether, these results reveal an unexpected mechanism involved in the cell cycle arrest upon infection: the CagA-dependent derepression of LATS2 by the downregulation of miR-372 and miR-373.

## Discussion

Recent data have highlighted the importance of miRNA in epithelial defense against pathogens such as *Cryptosporidium parvum *[18,19] or *Toxoplasma gondii *[40]. Gastric mucosa homeostasis is controlled by both immune and developmental mechanisms and its disruption by *H. pylori *infection can lead to severe inflammatory disorders or cancerous lesions of the stomach. While the impact of pathogens on the miRNA-mediated regulation of immune responses is beginning to be precisely understood [41], little is known about bacteria interfering with miRNAs regulating cell cycle and developmental pathways. In this study, we report that the bacterial pathogen *H. pylori *specifically downregulates an embryonic-specific miRNA cluster involved in the growth regulation of AGS cells, a non-polarized epithelial cell system commonly used to study *H. pylori*-host interactions. Through a deep-sequencing approach, we identified the whole content of miRNAs expressed in this gastric adenocarcinoma cell line. Among the 38 most expressed miRNAs, we found several miRNA previously described in gastric cancer, notably the miR-106b-93-25 cluster, the miR-221-222 cluster, miR-21 and let-7a [29,42,43]. However, we were surprised to discover that the most abundant of this repertoire, miR-372, belongs to a specific cluster encoding three other miRNAs, miR-371-5p, miR-371-3p and miR-373, expressed notably in human embryonic stem cells [34,44]. Interestingly these miRNAs were also mentioned in a miRNA profiling study in normal human tissues [35], in which they were found highly and specifically expressed in placenta, but also faintly detectable in the testis and stomach.

### Functional links of miR-371-372-373 cluster with miR-106b-93-25 and miR-221-222 in gastric cancer

miR-372 and miR-373 were initially discovered as novel oncogenes participating in the development of human testicular germ cell tumors by targeting the cell cycle inhibitor LATS2 [36]. Interestingly, this embryonic stem cell miRNA cluster shares some similarities with two miRNA clusters recently found to be overexpressed in gastric carcinomas, namely the miR-106b-93-25 cluster [43] and the miR-221-222 cluster [42]. In gastric cancers, these two clusters have been described to silence the CIP/KIP family members, p21^Cip1^, p27^Kip1 ^and p57^Kip2^, allowing the cell to overcome the G1/S checkpoint [42,43]. Surprisingly, these two clusters are poorly expressed in AGS cells as compared to other gastric cell lines and their expression levels are close to those observed in normal gastric mucosa ([42,43]; Cedric Belair and Cathy Staedel, unpublished results). One explanation is that in this cell line, the oncogenic role of these two clusters could have been replaced by the miR-371-372-373 cluster overexpression. Indeed, miR-372 and miR-373 belong to the miR-106b family which includes miR-93, miR-17-5p and miR-20a [45]. Members of this family harbor the same seed sequence, stressing that they might share similar functions. Recently, they have been shown to overcome the p21^Cip1^-induced cell cycle arrest observed during Ras-induced senescence [45]. In testicular germ cell tumors, which retained functional, wild-type p53, miR-372 and miR-373 act as oncogenes, silencing LATS2 and thus allowing cell proliferation [36]. Indeed, LATS2 is a serine/threonine kinase involved in a positive feedback loop with p53 [46] that can induce G1/S arrest by inhibiting cyclin E/cyclin-dependent kinase 2 (CDK2) kinase activity [47]. Interestingly, AGS cells express low wild-type p53 [48] and high p21^cip1/waf1 ^levels in basal growth conditions ([49]; Cedric Belair and Cathy Staedel, unpublished results). Thereby, as in the testicular germ cell tumors, abundant miR-372 and miR-373 in AGS cells, that totally silence LATS2, may participate in their active cell cycle progression despite high levels of p21^cip1/waf1^.

### *H. pylori *represses the miR-371-372-373 expression via its major virulence factor CagA

We report here that *H. pylori *specifically inhibits the synthesis of miR-372 and miR-373 at the primary transcript level and that this repression relies on CagA translocation in AGS cells through a functional T4SS. Very interestingly, these two miRNAs, already described to promote cell proliferation [36], have also been found to be repressed upon differentiation of embryonic stem cells in specific culture conditions [34]. In fact, *H. pylori*, via its major virulence factor CagA, can induce an intestinal transdifferentiation program in gastric epithelial cells that have undergone a cell cycle arrest [24]. In these cells, the proliferation is blocked at the G1/S transition after 24 h in the presence of *cag*PAI*+ H. pylori *and this stop is dependent on the translocation of CagA into the host cell [24,49]. Therefore, one could imagine that the observed repression of miR-372 and miR-373 in AGS cells could be a consequence of a block in cell division. However, at 2 h post infection, when cell proliferation has not been stopped yet, the pri-miR-371-372-373 was already downregulated close to its minimal level. This latter result suggests that the CagA-dependent repression of this cluster is a primary event and not a consequence of the cell cycle arrest. Further investigations may uncover the possible role of cell signaling pathways in this repression such as extracellular signal-regulated kinase (ERK), the phosphoinositide 3-kinase (PI3K)/Akt and notably the Src kinase, the activation of which occurs at this very initial stage of infection [50].

### The cell cycle arrest at the G1/S transition in AGS cells is mediated by the regulation of the couple miR-372-373/LATS2 protein

In this paper, we also reveal a new mechanism involved in the cell cycle arrest in AGS cells upon *H. pylori *infection. Indeed, we show that this bacterium induces a block in cell cycle progression at the G1/S transition via the post-transcriptional derepression of LATS2 synthesis. While several cell cycle regulators have been studied in this context, such as p21^cip1/waf1 ^[31,49,51], or p27^kip1 ^[52,53], our data indicate that an additional pathway can occur in AGS cells. Indeed, *H. pylori *is much less efficient in blocking cell proliferation when the level of LATS2 is maintained at a low level either by miR-372 and miR-373 mimics or by specific anti-LATS2 siRNAs. However, because the 3'UTR of p21^cip1/waf1 ^has also been proposed to be targeted by miR-372 and miR-373 [54], we certainly cannot exclude that its induction also participates in this cell cycle arrest [24,49]. However, the block at the G1/S transition in AGS cells requires the derepression of LATS2 synthesis via the repression of miR-372 and miR-373. Our results with the EGFP-LATS2 reporter cell line infected with type I strains, which harbor a functional *cag*PAI, clearly indicate that *H. pylori *induce a post-transcriptional release of LATS2 synthesis in a CagA-dependent manner through the downregulation of the miRNA cluster.

### AGS cells exhibit a stem cell miRNA signature

Because *cag*PAI increases the risk for infected patients to develop severe atrophic gastritis and distal intestinal-type gastric cancer [55], the perturbations of cell cycle progression elicited by *H. pylori *in gastric epithelial cells gave raise to many studies, some of which having precisely used AGS cells as a model system of an actively replicating gastric mucosa [31,51,56]. The miRNA content in AGS cells is characterized by its relative high abundance in embryonic stem cell miRNA [see Additional File 1, Table S2]. In fact, the growth advantages that AGS cells gained from the high expression of miR-371-372-373 cluster confer to them characteristics of gastric stem cell/progenitors rather than gastric mucosa differentiated epithelium. miR-373 expression has already been detected in stomach [33]. Epithelium turnover, which results from the balanced progenitor cell generation and differentiated cell death, is a major host defense mechanism against pathogens and its alteration is commonly observed upon bacterial infections [57]. Iwai and colleagues have shown that *Shigella flexneri *blocks cell cycle of intestinal progenitors through the injection of its bacterial effector IpaB [58]. In an animal model, the same group has demonstrated that *H. pylori *through CagA delivery suppresses apoptosis at superficial gastric pit cells, leading to delayed gastric epithelium self-renewal. Hence, it is tempting to speculate that *H. pylori *interferes with the gastric epithelium self-renewal repressing this specific embryonic miRNA cluster in gastric progenitors, an additional mechanism that Mimuro and coworkers did not observe [57].

## Conclusions

The role of miRNAs in severe pathologies, such as cancer, is now clearly established and extensively studied. Our work uncovers the unexpected pair of miR-372 and miR-373 as a novel example of miRNAs dysregulated in gastric cancer. Repressing LATS2 synthesis, through miR-372 and miR-373 overexpression, could represent an alternative pathway to the downregulation of p21^cip1/waf1 ^or p27^kip1 ^in the sequence of events leading to the malignant transformation of cells. We unveil LATS2/miR-372 and miR-373 as a novel mechanism in CagA-induced cell cycle arrest in proliferating gastric cells. Our findings enrich the library of cellular signaling perturbed by the Cag toxin with the miRNA pathway. Further studies will bring out the bacterial silencing repressor potential of CagA. Finally, our data are the first findings described so far showing a human pathogenic bacterium able to interfere with the biogenesis of a miRNAs cluster through the injection of its CagA toxin. However it is likely that it will be not the only one, as injection of toxin through a secretion system in order to interfere with host cell signaling pathways is a common virulence mechanism used by human pathogenic Gram-negative bacteria.

## Methods

### Cell culture

All the tissue culture reagents were purchased from Invitrogen (Paisley, UK ). The AGS gastric epithelial cell line (ATCC CRL 1739, Manassas, VA, USA) was maintained in Dulbecco's modified Eagle medium (DMEM)/F-12 (Ham's) medium, the other gastric epithelial cell line MKN-74 (HSRRB FC-2008-028, Osaka, Japan) in RPMI medium and the cervix cancer cell line (HeLa) in DMEM. All media were supplemented with 10% heat-inactivated fetal bovine serum (FBS), 2 mM l-glutamine and 1% non-essential amino acids. Cells were grown at 37°C in a humidified 5% CO_2 _atmosphere.

### *H. pylori *culture

*H. pylori *26695 strain (CIP 106780, Institut Pasteur, Paris, France) and its isogenic mutants, as well as P12 (generous gift of T. Meyer, MPI, Berlin, Germany), SS1 and X47-2AL (generous gift of H. de Reuse, Institut Pasteur, Paris, France) strains were grown on columbia agar plates supplemented with 7% laked horse blood, the Dent selective supplement (Oxoid, Basingstoke, UK), and 20 μg/ml kanamycine for the isogenic mutants, for 24 h at 37°C, in anaerobic jars, under microaerobic conditions (10% CO_2_, 6% O_2_) generated by CampyGen bags (Oxoid, Basingstoke, UK). After 24 h incubation, the bacteria were expanded onto fresh plates and grown for an additional 24 h. For infection, bacteria were harvested in complete Ham's medium and quantified by optical density at 600 nm, assuming that OD_600 _= 1 corresponded to 10^9 ^bacteria.

### Construction of *H. pylori *isogenic mutants

The isogenic knockout mutants 26695 Δ*cagA *and Δ*cagE *were constructed as previously described [59]. The genomic DNA of *H. pylori *strain 26695 and the vector pUC18K2 were used as template for all PCR amplifications in order to generate the kanamycine resistance cassette. [see Additional File 1, Table S4 for primers].

### Coculture of AGS cells with *H. pylori*

Before coculture, the culture medium was replaced with fresh and the bacterial suspension was added to AGS cells at 60% to 70% confluence at the indicated MOI. The cocultures were incubated at 37°C, in a humidified 5% CO_2 _atmosphere.

### RNA extraction

Total RNA was extracted using Trizol reagent (Invitrogen, Paisley, UK), according to the manufacturer's protocol. RNA concentrations were determined by a NanoDrop spectrophotometer (NanoDrop Technologies, Inc., Waltham, MA, USA). RNA quality was analyzed on a 2100 Bioanalyzer (Agilent Technologies, Santa Clara, CA, USA).

### 454 miRNA libraries

Libraries for '454' pyrosequencing of cDNA were constructed by *Vertis *Biotechnology AG, Freising, Germany (http://www.vertis-biotech.com/) as described previously for eukaryotic microRNA in brain [60] and murine macrophages [14]. Specifically, total RNA was prepared from AGS cells either grown in basal conditions or cocultured for 5 h with *H. pylori *26695 strain at MOI 240, and treated with DNase I. Small RNA species were isolated from DNase I treated RNA using the mirVana miRNA isolation kit (Ambion, Austin, Texas, USA). The small RNAs were separated on a denaturing 12.5% polyacrylamide (PAA) gel and stained with SYBRgreenII. As molecular mass standard, a mixture of oligonucleotides was used, also as internal size marker within the RNA samples. The population of miRNAs with a length of 19 to 29 nucleotides was obtained by passive elution of the RNAs from the gel. The miRNAs were then precipitated with ethanol and dissolved in 20 μl water, and 100 ng of RNA was used for cDNA synthesis. The RNA samples were poly(A)-tailed using poly(A) polymerase followed by ligation of a RNA adapter to the 5'-phosphate of the small RNAs. First-strand cDNA synthesis was then performed using an oligo(dT)-adapter primer and M-MLV H- reverse transcriptase. Incubation temperatures were 42°C for 20 min, ramp to 55°C followed by 55°C for 5 min. The resulting cDNAs were then PCR amplified using a high fidelity DNA polymerase. The primers used for PCR amplification were designed for amplicon sequencing according to the instructions of 454 Live Sciences and contain barcode sequences, which are attached to the 5'-ends of the cDNAs during PCR amplification. The following adapter sequences flank the cDNA inserts: 5'-end (54 bases) (5'-**CCATCTCATCCCTGCGTGTCTCCGACTCAG**-NNNNNN-GACCTTGGCTGTCACTCA-3) and 3'-end (84 bases) (5'-**CCTATCCCCTGTGTGCCTTGGCAGTCTCAG**-ATCAGGCAGAGGACGAGACATCGCCCCGC(dT25)-3'). The 454 adapter sequences are in bold.

The combined length of the flanking sequences was 104 bases. Therefore, PCR products containing miRNA sequences of 19 to 29 nucleotides must have a total length of about 120 to 135 bp. PAGE analysis of the PCR-amplified cDNAs revealed that the cDNAs were of the expected size. The 120 to 135 bp fractions of the cDNAs were obtained by separation of the cDNAs on preparative 6% PAA gels. The eluted cDNAs were finally extracted with phenol/chloroform and precipitated with ethanol. DNA pellet was dissolved in 30 μl water. Concentration of the samples was about 10 ng/μl. The resulting cDNA libraries were sequenced on a Roche GS20 454 sequencer at the MPI for Molecular Genetics in Berlin, Germany.

### miRNA sequencing analysis

The 454 reads were aligned against the respective set of mature miRNAs using the R package mirMap454. Briefly, mirMap454 attempts to identify common 5' and 3' adapter sequences in the reads and removes these prior to further analysis. Each read is aligned against all mature miRNAs in the database semiglobally using dynamic programming. The simple scoring scheme employed allows terminal gaps in the mature miRNA sequence at no costs, while indels in general are scored with +3, mismatches with +2 and mismatches to N with +1. Motivated by the error distribution of 454 reads, up to three terminal mismatches are ignored in scoring the alignment if the remainder of the sequences aligns without indels or mismatches. For each read, the alignment to a mature miRNA with lowest score is identified. If the score of this alignment is 5 or less, the read is considered to be mapped to this miRNA. If the read maps to n different miRNAs with equal scores, 1/n reads are counted as mapped to each of these miRNAs [see Additional File 1, Table S1 for results].

### Quantitative RT-PCR

TaqMan microRNA assays (Applied Biosystems, Carlsbad, CA, USA) were used to quantify the expression of mature miR-371-3p (AB 002124), miR-372 (AB 000560) and miR-373 (AB 000561). Real-time RT-qPCR was performed in triplicate on 20 ng total RNA according to the manufacturer's instructions. miR-372 copy number was determined by absolute RT-qPCR. Sample Ct value was compared to a standard curve determined using serially diluted synthetic miR-372 [see Additional File 1, Table S4] and expressed as copies per cell on the basis of our determination of 1 μg RNA/10^5 ^AGS cells. LATS2 mRNA and pri-miR-371-372-373 levels were determined by RT-qPCR using PerfeCta SYBR Green SuperMix (Quanta BioSciences, Gaithersburg, MD, USA). cDNA was synthesized with Superscript II reverse transcriptase (Invitrogen, Paisley, UK) using oligo(dT)_20 _primer according to the manufacturer's protocol [see Additional File 1, Table S4 for qPCR primers]. miRNAs levels were normalized to U6 small nuclear RNA (snRNA) (RNU6b, AB 001093) or to SNORD49A (RNU49, AB 001005) in infection experiments, because RNU6b was found affected by the infection. The levels of LATS2 mRNA or pri-miR-371-372-373 were normalized to the ribosomal protein P0 mRNA. Relative expressions were calculated using the comparative Ct method.

### Northern blots

Total RNA (20 μg) were resolved on a 15% denaturing PAA gel and blotted onto nylon membranes (Hybond-N, GE Healthcare, Chalfont St. Giles, UK). Membranes were incubated with 5'-^32^P-radiolabeled locked nucleic acid (LNA)/DNA probes in 50% formamide, 5 × SSPE (Saline-Sodium Phosphate-EDTA), 5 × Denhardt solution, 0.5% SDS, 20 μg/ml salmon sperm DNA, at 42°C overnight in a hybridization oven. After two washes for 5 min each in 2 × SSC (Saline-Sodium Citrate), 0.1% SDS at 42°C, the blots were analyzed and quantified by phosphorimaging (Molecular Imager PharosFX^plus^, BioRad, Hercules, CA, USA) [see Additional File 1, Table S4 for probe sequences].

### Western blot

Cells were harvested in ice-cold Ham's medium and washed with ice cold phosphate-buffered saline (PBS). The cell pellets were lysed in ProteoJET Mammalian Cell lysis reagent (Fermentas, St. Leon-Rot, Germany) supplemented with 5 mM ethylenediaminetetraacetic acid (EDTA) and 1 × ProteoBlock Protease inhibitor cocktail (Fermentas, St. Leon-Rot, Germany). Proteins were separated by SDS-PAGE and western blot was performed using Immobilon-P transfer membrane (Millipore), according to standard procedures. Anti-LATS2 (clone ST-3D10, Abnova, Taipei City, Taiwan) and anti-α-tubulin (clone B-5-1-2, Sigma-Aldrich, St. Louis, MO, USA) antibodies were used at a 1:500 and 1:30,000 dilutions, respectively.

### Cell transfections

All transfections were performed using Lipofectamine 2000 (Invitrogen, Paisley, UK) according to the manufacturer's protocol [see Additional File 1, Table S4 for oligonucleotide sequences]. Double-stranded mimic 372-373 were generated by incubating equimolar amounts of complementary, heat-denatured single RNA strands in 60 mM KCl, 6 mM 4-(2-hydroxyethyl)-1-piperazineethanesulfonic acid (Hepes) pH 7.5, 0.2 mM MgCl_2 _buffer, for 20 min at room temperature. Anti-human LATS2 siRNA (siLATS2) and control siRNA were purchased from Qiagen (Courtaboeuf, France). AGS cells were transfected twice with 100 nM of miRNA-antisense (as372-373), 100 nM scrambled oligonucleotides (sc372-373), or 25 nM double-stranded mimics, 20 nM siLATS2 or 20 nM control siRNA. pCMVmyc-LATS2 (generous gift of Professor H Nojima, Osaka, Japan) or the empty vector pcDNA3 (Invitrogen, Paisley, UK) were transfected as indicated in the figure legends. NFκB activation was monitored using the BD Mercury firefly luciferase reporters (BD Biosciences, Franklin Lakes, NJ, USA) according to the manufacturer's instructions. Briefly, 48 h before infection, cells were transfected with either 1 μg NFκB-luc or TAL (luciferase without promoter) vectors, together with 10 ng of pRL-SV40 control vector (Promega, Madison, WI, USA). LATS2 translation efficiency was assessed with the luciferase sensors pGL3-LATS2 or its mutant pGL3-LATS2mut [34] (generous gifts of Professor R Agami, The Netherlands Cancer Institute, Amsterdam, The Netherlands). Each plasmid was transfected at 100 ng in the presence of 10 ng pRL-SV40 control vector (Promega, Madison, WI, USA). At the second transfection round with as372-373 or sc372-373, the oligonucleotides were mixed with 100 ng pGL3-LATS2 or pGL3 plasmids, and 10 ng pRL-SV40 control vector. Firefly and *Renilla *luciferases were measured 48 h post transfection using the Dual Luciferase Assay (Promega, Madison, WI, USA). Firefly luciferase activities were normalized for transfection efficiency by *Renilla *luciferase.

### Construction of the LATS2 reporter AGS cell line

The LATS2 3'UTR sequence was obtained by PCR using pGL3-LATS2 as template [see Additional File 1, Table S4 for primers]. The PCR product was cloned into the pEGFP-C1 vector (Clontech, Saint-Germain-en-Laye, France) downstream to the *egfp *gene. Stable LATS2 reporter AGS cells were obtained after selection with 400 μg/ml geneticin (Invitrogen, Paisley, UK ) of AGS cells transfected with the pEGFP-3'UTR-LATS2 vector.

### Cell cycle analysis by flow cytometry

After coculture with *H. pylori*, adherent and floating cells were collected together, washed once in ice-cold PBS and fixed in 70% ice-cold ethanol for 24 h. Fixed cells were stained in a 0.5 mg/ml propidium iodide, 0.1% (w/v) RNAse A, 0.1% (w/v) bovine serum albumin (BSA)/PBS solution and analyzed for DNA content by flow cytometry (FACS Canto cytometer, BD Biosciences, Franklin Lakes, NJ, USA).

### BrdU incorporation

AGS cells were transfected twice with as372-373, sc372-373 or mimic 372-373 before coculture with *H. pylori*. Cells were pulse labeled with 10 nM BrdU for 1 h before the end of the coculture. Cells were harvested and BrdU incorporation was detected by immunofluorescent staining using the fluorescein isothiocyanate (FITC) BrdU Flow kit (Biosciences, Franklin Lakes, NJ, USA) and quantified by flow cytometry (FACS Canto cytometer, BD Biosciences, Franklin Lakes, NJ, USA).

## Competing interests

The authors declare that they have no competing interests.

## Authors' contributions

CB carried out the cell and *Helicobacter pylori *cultures, transfections and miRNA and target analyses. JB carried out the *H. pylori *cultures, generated the mutants, and participated to the miRNA and target analyses. SC designed and purified the oligonucleotides and performed the northern blots. The '454' high-throughput pyrosequencing was performed and analyzed by CMS under JV's supervision. CS and FD conceived the study, supervised CB and JB's work. FD, CB and CS wrote the manuscript. All authors read and approved the manuscript.

## Supplementary Material

Additional file 1 **Tables S1-4**. Table S1: 454 results of the micro RNA (miRNA) content in AGS cells in basal conditions and upon *Helicobacter pylori *infection (MirBase 14.0). miRNAs studied in this paper are represented in bold. *P *values are calculated using Fisher's exact test. Table S2: miRNAs listed in regards of their known function. miRNAs counting more than 100 reads (>0.4% reads) correspond to those in Figure 1. Table S3: *H. pylori *strains used in this study. Table S4: Oligonucleotides used in this study. Sequences are given in the 5' to 3' direction. For locked nucleic acid (LNA) oligonucleotides small letters indicate DNA, whereas capital letters indicate LNA. Antisense miRNA is abbreviated as number of the miRNA. Sequences homologous to the *aphA-3' *kanamycin resistance cassette gene are shown in italics, lower case letters. Click here for file

Additional file 2 **Figure S1**. Large tumor suppressor homolog 2 (LATS2) translation efficiency in AGS (high miR-372 and miR-373) and MKN-74 (low miR-372 and miR-373) cells. AGS or MKN-74 cells were plated at 0.7.10^5 ^or 10^5 ^cells/well, respectively, in a 24-well plate and transfected the following day with 100 ng/well pGL3, pGL3-LATS2 or pGL3-LATS2mut vectors [36], each mixed with 10 ng pRL-SV40 vector. Luciferase activities were measured 48 h post transfection. Bars indicate the relative firefly luciferase activity normalized to the *Renilla *activity and compared to pGL3. Data are mean ± SD from three independent experiments in triplicate. *P < 0.05; ***P < 0.001. Click here for file

Additional file 3 **Figure S2**. Effect of antisense oligonucleotides treatment on micro RNA (miRNA) expression **(A,B)**, large tumor suppressor homolog 2 (LATS2) expression **(D) **and cell growth rate **(C,E)**. (A) Endogenous expression of miR-372, miR-373 and miR-200b was determined 48 h post transfection by quantitative real time PCR (RT-qPCR) in untreated cells (mock) or cells treated twice with 100 nM of locked nucleic acid (LNA)/DNA antisense (as372-373) or scramble (sc372-373) oligonucleotides [see Additional File 1, Table S4 for sequences]. Bars indicate relative miRNA expression normalized to U6 small nuclear RNA (snRNA) (RNU6b). Data are mean ± SD of two independents experiments. (B) Northern blot analysis of untreated cells (lane 2) or cells treated with as372 (lane 3), as373 (lane 4), the mix as372-373 (lane 5) or the mix sc372-373 (lane 6). M: DNA (bp) marker lane (Promega). (C) Comparison of AGS growth rate between non-treated (mock) or cells treated with antisense (as372-373, as200b-200c) or scrambled (sc372-373, sc200b-200c) oligonucleotides. Cells were plated at a density of 10^5 ^cells per well in a six-well plate and counted at day 2 and day 3 after plating. The results are expressed as the relative increase in cell numbers between day 2 and day 3, normalized to that of untreated AGS cells. Data are mean ± SD of three independent experiments in duplicate. (D) LATS2 immunoblot of AGS cells transfected with a pCMVmycLATS2 expression vector, or with as 372-373 or sc372-373 oligonucleotides. A total of 10^6 ^AGS cells were plated in 6 cm diameter plates and transfected the following day with 1 μg pCMVmycLATS2. Oligonucleotide treatments were performed as above. Proteic lysates were prepared 48 h post transfection and submitted to SDS-PAGE. α-Tubulin was used as a loading control. A representative immunoblot is shown. (E) Effect of ectopic expression of LATS2 on AGS or MKN74 growth rate. Black bars, cells transfected with pCMVmycLATS2; grey bars, cells transfected with pcDNA3. Cells were plated at a density of 10^3 ^cells/well in a 96-well plate containing 22 ng/well plasmid. Cell viability was determined at day 2 and day 3 after plating by optical density later using the Celltiter Proliferation Assay (Promega). The results are expressed as the relative increase in optical density between day 2 and day 3, normalized to that of pcDNA3-transfected cells. Data are mean ± SD of two independent experiments performed in triplicate. Click here for file

Additional file 4 **Figure S3**. *Helicobacter pylori-*induced morphological changes **(A) **and proinflammatory responses **(B,C) **in AGS cells. (A) Morphology of uninfected AGS cells or cells infected for 24 h with *H. pylori *26695 wild-type strain or its isogenic mutants *H. pylori *ΔCagA and ΔCagE at multiplicity of infection (MOI) 100 was visualized by phase contrast microscopy. Uninfected AGS cells display an epithelial morphology. Coculture with *H. pylori *26695 wild-type strain causes cell elongation, so called 'hummingbird' phenotype, and vacuoles. The 'hummingbird' phenotype is CagA dependent, as cells cocultured with *H. pylori *26695 mutated for the CagA toxin (*H. pylori *ΔCagA) or unable to inject CagA (*H. pylori *ΔCagE) harbor only vacuoles, an effect due to the VacA toxin secreted into the culture medium. (B,C) Proinflammatory responses of AGS cells infected for 24 h with *H. pylori *26695 wild-type strain or its isogenic mutants ΔCagA and ΔCagE at MOI 100 were analyzed by nuclear factor (NF)κB activation (B) and interleukin 8 (IL-8) secretion (C). As previously described [26], NFκB activation and IL-8 secretion were both CagA and Type IV Secretion System dependent. NFκB activation was monitored using the BD Mercury firefly luciferase reporters (Beckton-Dickinson) containing NFκB binding sites upstream of the firefly luciferase. Bars indicate the firefly luciferase activity normalized to *Renilla *luciferase, minus TAL (luciferase without promoter) activity. Data represent the mean ± SD of two independent experiments. IL-8 secreted in the culture medium was measured 24 h post infection using ELISA (R&D systems). The results represent the mean ± SD of four independent experiments. Click here for file

Additional file 5 **Figure S4**. Enhanced green fluorescent protein (EGFP) fluorescence of large tumor suppressor homolog 2 (LATS2) reporter-AGS cells transfected with as372-373 or sc372-373. Cells were transfected twice with 100 nM antisense or scramble oligonucleotides and observed 24 h after the last transfection on an inverted microscope (Zeiss) in phase contrast (right panels) or epifluorescence (left panels). Click here for file

Additional file 6 **Figure S5**. *Helicobacter pylori *strain-specific repression of primary (pri-)miR-371-372-373 and upregulation of the enhanced green fluorescent protein (EGFP)-3' untranslated region (UTR) large tumor suppressor homolog 2 (LATS2) reporter gene. AGS cells were infected with *H. pylori *type I strains (P12) or type II strain (SS1, X47) at a multiplicity of infection (MOI) 100 for 24 h. **(A) **Expression of the pri-miR-371-372-373 was determined by quantitative real time PCR (RT-qPCR) in non-infected cells (NI) or cells infected with the different *H. pylori *strains. Bars indicate relative pri-micro RNA (miRNA) expression normalized to ribosomal protein P0. A representative experiment is shown. **(B) **EGFP fluorescence of LATS2 reporter cells infected or not with *H. pylori *strains. Cells were observed 24 h post infection on an inverted microscope (Zeiss) in phase contrast (right panels) or epifluorescence (left panels). Click here for file

Additional file 7 **Figure S6**. CagA-dependent accumulation of *Helicobacter pylori*-infected AGS cells in G0/G1 phase. Cellular DNA contents of uninfected AGS cells or cells infected for 24 h with wild-type, ΔCagA or ΔCagE *H. pylori *were stained by iodide propidium and analyzed by flow cytometry. Click here for file
