# Supplementary material for: Helicobacter pylori interferes with an embryonic stem cell micro RNA cluster to block cell cycle progression
Source: Silence. 2011 Oct 25;2:7. doi: 10.1186/1758-907X-2-7 (PMC3212895; doi:10.1186/1758-907X-2-7)
Supplement: Additional file 1 — Tables S1-4. Table S1: 454 results of the micro RNA (miRNA) content in AGS cells in basal conditions and upon Helicobacter pylori infection (MirBase 14.0). miRNAs studied in this paper are represented in bold. P values are calculated using Fisher's exact test. Table S2: miRNAs listed in regards of their known function. miRNAs counting more than 100 reads (>0.4% reads) correspond to those in Figure 1. Table S3: H. pylori strains used in this study. Table S4: Oligonucleotides used in this study. Sequences are given in the 5' to 3' direction. For locked nucleic acid (LNA) oligonucleotides small letters indicate DNA, whereas capital letters indicate LNA. Antisense miRNA is abbreviated as number of the miRNA. Sequences homologous to the aphA-3' kanamycin resistance cassette gene are shown in italics, lower case letters. [file 1758-907X-2-7-S1.PDF]

Table S1: 454 results of the miRNA content in AGS cells in basal conditions and upon *H. pylori* infection (MirBase 14.0).

MiRNA studied in this paper are represented in bold. *P*-values are calculated using Fisher's exact test.

	Number of reads		
**MiRNA name**	AGS	AGS + Hp	*P*-value
**hsa-miR-372**	**2814**	**1595**	**5.1075 x 10^{-13}**
hsa-miR-27b	1735.5	1057.5	0.00020114
hsa-miR-200b	1564	1211	0.00800858
hsa-miR-23a	1202	1115	5.7139 x 10^{-12}
hsa-miR-92a	1117	709	0.03455711
hsa-miR-23b	1071	920	4.3226 x 10^{-6}
hsa-miR-200c	1007	1014	9.8946 x 10^{-17}
hsa-miR-17	978.333313	738.333374	0.12693797
hsa-miR-16	971	662	0.5729942
**hsa-miR-371-5p**	**919**	**562**	**0.00916823**
hsa-miR-20a	896.333313	602.333313	0.42291853
**hsa-miR-371-3p**	**558**	**433**	**0.1170863**
hsa-let-7a	557.5	590.5	2.3337 x 10^{-12}
hsa-miR-15b	533	465	0.00055788
hsa-miR-103	518.5	249	4.6014 x 10^{-7}
hsa-miR-107	489.5	234	8.3897 x 10^{-7}
hsa-miR-93	458	258	0.00458602
hsa-miR-21	447	312	0.97033239
hsa-let-7f	392.5	322.5	0.03191492
hsa-miR-25	391	302	0.21319636
hsa-miR-27a	388.5	292.5	0.36659655
hsa-miR-320d	362.666687	166.666672	3.6934 x 10^{-6}
hsa-miR-320c	336.666687	150.666672	2.7853 x 10^{-6}
**hsa-miR-373**	**331**	**137**	**8.3668 x 10^{-8}**
hsa-miR-151-5p	265	178	0.66276123
hsa-miR-106b	255.5	183	0.84559503
hsa-miR-361-5p	252	172	0.80434631
hsa-miR-1297	221	168	0.4379612
hsa-let-7b	215	182.5	0.06510962
hsa-miR-24	195	145	0.61872525
hsa-let-7d	193	189	0.00118367
hsa-miR-26a	164	113.5	0.9026762
hsa-miR-125a-5p	160	80	0.01247193

hsa-miR-1308	155	199	1.3647×10^{-8}
hsa-miR-99b	129	70	0.08366475
hsa-miR-7	123	57	0.00972107
hsa-let-7g	122	131	0.00083166
hsa-miR-191	121	74	0.38182701
hsa-miR-106a	117.333336	91.3333359	0.48037254
hsa-miR-222	117	63	0.09514082
hsa-miR-320a	116.666664	29.6666679	1.2526×10^{-7}
hsa-miR-374b	113	138	1.1196×10^{-5}
hsa-miR-15a	94	41	0.01089894
hsa-miR-203	93	58	0.50840016
hsa-miR-1827	93	92.5	0.01675523
hsa-miR-22	84	60	0.93253546
hsa-let-7e	79.5	69.5	0.18197681
hsa-miR-19b	77	19	1.0072×10^{-5}
hsa-miR-221	66	39	0.42801578
hsa-miR-182	65	18	0.00020488
hsa-miR-30b	65	34	0.18408001
hsa-miR-30d	63	37	0.41711646
hsa-miR-532-3p	63	34	0.25591646
hsa-miR-34a	60	34	0.34621697
hsa-miR-194	59	46	0.62017894
hsa-miR-151-3p	50	45	0.25106272
hsa-miR-1307	50	28	0.35921821
hsa-miR-30c	44	29	0.81332648
hsa-miR-26b	43	49.5	0.01523789
hsa-miR-378	43	18	0.06861116
hsa-miR-185	40	20	0.23882918
hsa-miR-29a	38	29	0.80404246
hsa-miR-423-3p	36	23	0.79199639
hsa-miR-532-5p	36	23	0.79199639
hsa-miR-141	34	16	0.19857956
hsa-miR-1979	33	13	0.09815114
hsa-miR-148a	27	27	0.21353774
hsa-miR-190	27	11	0.1395019
hsa-miR-425	26	13	0.33475349
hsa-miR-183	25	27	0.12270808
hsa-miR-455-3p	24	8	0.07233645
hsa-miR-720	23	10	0.22044175
hsa-miR-30e	21	9	0.26633076
hsa-miR-130b	20	3	0.00522039
hsa-miR-502-3p	20	9	0.34587705
hsa-miR-590-3p	18	9	0.44123845

hsa-miR-10a	17	4	0.04532705
hsa-miR-195	16	12	0.85033946
hsa-miR-374a	16	12	0.85033946
hsa-miR-28-5p	15	17	0.20840921
hsa-let-7i	15	12	0.84540483
hsa-miR-18a	14	4	0.14905045
hsa-miR-192	14	9	1
hsa-miR-92b	14	4	0.14905045
hsa-miR-140-3p	13	6	0.48801277
hsa-miR-429	13	8	0.82817109
hsa-miR-652	13	14	0.32829731
hsa-miR-421	13	17	0.09615495
hsa-miR-365	12	10	0.67243146
hsa-miR-501-3p	12	4	0.21416522
hsa-miR-942	12	4	0.21416522
hsa-miR-28-3p	11	12	0.29679702
hsa-miR-31	11	6	0.80635255
hsa-miR-135b	11	6	0.80635255
hsa-miR-484	11	7	1
hsa-miR-497	11	1	0.01965782
hsa-miR-500	11	4	0.30286431
hsa-miR-886-5p	11	6	0.80635255
hsa-miR-98	10	16	0.04509122
hsa-miR-301a	10	2	0.13943931
hsa-miR-362-5p	10	6	0.80620105
hsa-miR-362-3p	10	7	1
hsa-miR-96	9	1	0.05453144
hsa-miR-128	9	16	0.02492443
hsa-miR-130a	9	6	1
hsa-miR-1274b	9	2	0.13885849
hsa-miR-101	8	7	0.79432724
hsa-miR-210	8	13	0.07409052
hsa-miR-324-3p	8	8	0.61278049
hsa-miR-345	8	1	0.09109875
hsa-miR-1275	8	10	0.23737726
hsa-miR-1280	8	4	0.7714738
hsa-miR-186	7	3	0.53945125
hsa-miR-330-3p	7	5	1
hsa-miR-1301	7	3	0.53945125
hsa-miR-454	7	3	0.53945125
hsa-miR-1975	7	0	0.04643186
hsa-miR-215	6	7	0.40474737
hsa-miR-200a	6	6	0.56783778
hsa-miR-361-3p	6	6	0.56783778
hsa-miR-339-5p	6	1	0.25175261

hsa-miR-671-5p	6	3	0.74471826
hsa-miR-744	6	1	0.25175261
hsa-miR-302b	5.66666651	1.83333337	0.48349435
hsa-miR-132	5	3	1
hsa-miR-1303	5	4	1
hsa-miR-1268	5	3	1
hsa-miR-19a	4	1	0.65491635
hsa-miR-29c	4	1	0.65491635
hsa-miR-324-5p	4	0	0.14820995
hsa-miR-486-5p	4	6	0.33572563
hsa-miR-146b-5p	4	2	1
hsa-miR-574-3p	4	3	1
hsa-miR-615-3p	4	2	1
hsa-miR-877	4	3	1
hsa-miR-302e	3.66666627	1.33333337	0.65491516
hsa-miR-302f	3.66666627	1.33333337	0.65491516
hsa-miR-196a	3	2	1
hsa-miR-197	3	7	0.10382156
hsa-miR-125a-3p	3	1	0.64773039
hsa-miR-149	3	3	0.69576509
hsa-miR-574-5p	3	0	0.2730918
hsa-miR-584	3	3	0.69576509
hsa-miR-1226	3	0	0.2730918
hsa-miR-1261	3	3	0.69576509
hsa-miR-218	2	0	0.51539453
hsa-miR-140-5p	2	1	1
hsa-miR-296-3p	2	0	0.51539453
hsa-miR-375	2	0	0.51539453
hsa-miR-148b	2	3	0.40893409
hsa-miR-331-3p	2	1	1
hsa-miR-423-5p	2	4	0.23774553
hsa-miR-486-3p	2	0	0.51539453
hsa-miR-501-5p	2	0	0.51539453
hsa-miR-502-5p	2	0	0.51539453
hsa-miR-505	2	1	1
hsa-miR-573	2	0	0.51539453
hsa-miR-548c-5p	2	1	1
hsa-miR-660	2	0	0.51539453
hsa-miR-935	2	4	0.23774553
hsa-miR-519c-5p	1.5	0.5	1
hsa-miR-519b-5p	1.5	0.5	1
hsa-miR-32	1	0	1
hsa-miR-181b	1	0	1
hsa-miR-199b-5p	1	0	1
hsa-miR-204	1	0	1

hsa-miR-219-5p	1	0	1
hsa-miR-152	1	0	1
hsa-miR-184	1	1	1
hsa-miR-302a	1	0	1
hsa-miR-330-5p	1	0	1
hsa-miR-331-5p	1	2	0.57274506
hsa-miR-339-3p	1	4	0.16689864
hsa-miR-335	1	2	0.57274506
hsa-miR-193b	1	2	0.57274506
hsa-miR-181d	1	0	1
hsa-miR-518b	1	0	1
hsa-miR-455-5p	1	1	1
hsa-miR-550	1	0	1
hsa-miR-624	1	0	1
hsa-miR-628-5p	1	1	1
hsa-miR-641	1	0	1
hsa-miR-642	1	2	0.57274506
hsa-miR-643	1	0	1
hsa-miR-767-5p	1	0	1
hsa-miR-1283	1	0	1
hsa-miR-874	1	0	1
hsa-miR-301b	1	0	1
hsa-miR-1180	1	0	1
hsa-miR-1246	1	3	0.31291113
hsa-miR-1257	1	0	1
hsa-miR-1259	1	0	1
hsa-miR-1274a	1	2	0.57274506
hsa-miR-548i	1	1	1
hsa-miR-664	1	2	0.57274506
hsa-miR-2110	1	0	1
hsa-miR-2277	1	2	0.57274506
hsa-miR-2278	1	1	1
hsa-let-7c	0.5	4.5	0.02887547
hsa-miR-20b	0.5	0	1
**Total (reads)**	25348	17778.5	

Table S2: miRNA listed in regards of their known function.
MiRNA counting more than 100 reads (>0.4% reads) correspond to those of Figure 1.

**Hypoxia**		
**miRNA**	**Number of reads in AGS cells**	**References**
miR-23a	1202	[1, 2]
miR-92a	1117	[1-3]
miR-17	978	[1-3]
miR-20a	896	[1-3]
miR-21	447	[1, 2]
miR-373	331	[1, 2, 4]
miR-26a	164	[1, 2]
miR-191	121	[1, 2]
miR-19b	77	[1-3]
miR-30d	63	[1, 2]
miR-18a	14	[1-3]
miR-192	14	[1, 2]
miR-210	8	[1, 2, 5]
miR-200a	6	[1, 2]
miR-19a	4	[1-3]
miR-181b	1	[1, 2]
**Total (reads)**	**5443**	
**Total (%)**	**21**	

**Stem cell signature**		
**Embryonic stem cell**		
**miRNA**	**Number of reads in AGS cells**	**References**
miR-372	2814	[6, 7]
miR-200c	1007	
miR-371-5p	919	
miR-371-3p	558	
miR-21	447	
miR-373	331	
miR-222	117	
miR-374b	113	
miR-374a	16	
miR-301a	10	
miR-301b	1	
**Sum (reads)**	**6333**	
**Sum (%)**	**25.5**	
**Somatic stem cells**		
**miRNA**	**Number of reads in AGS cells**	**References**
miR-17	978	[6, 7]
miR-16	971	
miR-20a	896	
miR-103	518	

miR-107	489	
miR-24	195	
miR-106a	117	
miR-128	9	
miR-181b	1	
**Sum (reads)**	**4174**	
**Sum (%)**	**16.5**	
**Total (reads)**	**10507**	
**Total (%)**	**41.4**	

Table S3: *H. pylori* strains used in this study.

Name	Origin	Characteristics	References
CIP 106780	CIP Pasteur	26695 sequenced reference strain	[8]
CagA-1	derived of 26695 strain	HP0547::*aphA3'*; Km^R	this study
CagE-1	derived of 26695 strain	HP0544::*aphA3'*; Km^R	this study
P12	T. Meyer	P12 sequenced reference strain	Laboratory stock
SS1	H. de Reuse		Laboratory stock
X47-2AL			Laboratory stock

Table S4: Oligonucleotides used in this study.

Sequences are given in 5′→3′ direction. For LNA antisenses small letters indicate DNA, whereas capital letters indicate LNA. Antisense miRNA is abbreviated as asnumber of the miRNA. Sequences homologous to the *aphA-3′* kanamycin resistance cassette gene are shown in italics, lower case letters.

Oligo	Sequence	Used for	Gene	Name	References
FD142	*gttagtcacccgggtac*GTGTCTTTGAACATTCTTATT	Mutant construction (Rev)	HP0547	cagA	This study
FD143	CAAGCAAAAACAAACCCAAGCTGATCAGAG	Mutant construction (Fwd)	HP0547	cagA	
FD144	*tacctggagggaataatg*GGATTAAGGAATACCAAAAAC	Mutant construction (Fwd)	HP0547	cagA	
FD145	CTTAGGATCGTAAAATTGCGAGGTATTTTTG	Mutant construction (Rev)	HP0547	cagA	
FD146	CGACGCTTTTGTTTGGTAAC	Mutant construction (Fwd)	HP0547	cagA	
FD152	*gttagtcacccgggtac*CCCTCTCTTTATAGATATACC	Mutant construction (Rev)	HP0544	cagE	
FD153	GAAAAAAACACTCCCAATGGGCTTGTTTAT	Mutant construction (Fwd)	HP0544	cagE	
FD154	*tacctggagggaataatg*CAAAGCATCACTGATCTTTTG	Mutant construction (Fwd)	HP0544	cagE	
FD155	GAAAAACTATCCAAAATCGTCAAGACATCA	Mutant construction (Rev)	HP0544	cagE	
FD156	GTAATAAAAAAACTGAGAGGC	Mutant construction (Fwd)	HP0544	cagE	
FD70	GTACCCGGGTGACTAACTAGG	Mutant construction (Fwd)	*aphA3'*	Kanamycin	
FD71	CATTATTCCCTCCAGGTACTA	Mutant construction (Rev)	*aphA3'*	Kanamycin	
F-LATSUTR	AAAACTCGAGCTTAGATGGGGGCCAGGCACCCCCA	EGFP-3'UTR LATS2 construction			
R-LATSUTR	AAAAGGATCCCATTATTGCACAGAGATTTCTCATCAATGT				
CS60	acGctCaaAtgTcgCagCacTtt	LNA Northern blot probe and as372		miR-372	
CS61	acAccCcaAaaTcgAagCacTtc	LNA Northern blot probe and as373		miR-373	
CS16	CACGAATTTGCGTGTCATCCTT	LNA Northern blot probe	*RNU6-1*	U6 snRNA	
CS63	caCgtAcaTagTgcAccGatCtt	LNA antisense control		scramble miR-372	
CS64	caCacCacAatAgcAacGcaTtc			scramble miR-373	
3Fw-primiR371	AGTTTGCAGTGAGCCGAGAT	qPCR		primiR-371-372-373	
3Rev-primiR371	ATACAGCCCCTTGGTCACAG			primiR-371-372-373	
LATS2-Rev	CCCGCACAATCTGCTCATTC		*LATS2*	LATS2	[9]
LATS2-Fw	CAGGATGCGACCAGGAGATG				
Fw-P0	GCGACCTGGAAGTCCAACT		*RPLP0*	P0	This study
Rev-P0	CCATCAGCACCACAGCCTTC				
372-RNA	AAAGUGCUGCGACAUUUGAGCGU	mimic 372	*hsa-miR-372*	miR-372	This study
372*-RNA	CUCAAAUGUGGAGCACUAUUCU		*hsa-miR-372**	miR-372*	
373-RNA	GAAGUGCUUCGAUUUUGGGGUGU	mimic 373	*hsa-miR-373*	miR-373	
373*-RNA	ACUCAAAAUGGGGGCGCUUUCC		*hsa-miR-373**	miR-373*	

References

1. Kulshreshtha R, Ferracin M, Wojcik SE, Garzon R, Alder H, Agosto-Perez FJ, Davuluri R, Liu CG, Croce CM, Negrini M, et al: **A microRNA signature of hypoxia.** *Mol Cell Biol* 2007, **27:**1859-1867.
2. Guimbellot JS, Erickson SW, Mehta T, Wen H, Page GP, Sorscher EJ, Hong JS: **Correlation of microRNA levels during hypoxia with predicted target mRNAs through genome-wide microarray analysis.** *BMC Med Genomics* 2009, **2:**15.
3. Yan HL, Xue G, Mei Q, Wang YZ, Ding FX, Liu MF, Lu MH, Tang Y, Yu HY, Sun SH: **Repression of the miR-17-92 cluster by p53 has an important function in hypoxia-induced apoptosis.** *Embo J* 2009, **28:**2719-2732.
4. Crosby ME, Kulshreshtha R, Ivan M, Glazer PM: **MicroRNA regulation of DNA repair gene expression in hypoxic stress.** *Cancer Res* 2009, **69:**1221-1229.
5. Mathew LK, Simon MC: **mir-210: a sensor for hypoxic stress during tumorigenesis.** *Mol Cell* 2009, **35:**737-738.
6. Suh MR, Lee Y, Kim JY, Kim SK, Moon SH, Lee JY, Cha KY, Chung HM, Yoon HS, Moon SY, et al: **Human embryonic stem cells express a unique set of microRNAs.** *Dev Biol* 2004, **270:**488-498.
7. Gangaraju VK, Lin H: **MicroRNAs: key regulators of stem cells.** *Nat Rev Mol Cell Biol* 2009, **10:**116-125.
8. Tomb JF, White O, Kerlavage AR, Clayton RA, Sutton GG, Fleischmann RD, Ketchum KA, Klenk HP, Gill S, Dougherty BA, et al: **The complete genome sequence of the gastric pathogen Helicobacter pylori.** *Nature* 1997, **388:**539-547.
9. Voorhoeve PM, le Sage C, Schrier M, Gillis AJ, Stoop H, Nagel R, Liu YP, van Duijse J, Drost J, Griekspoor A, et al: **A genetic screen implicates miRNA-372 and miRNA-373 as oncogenes in testicular germ cell tumors.** *Cell* 2006, **124:**1169-1181.
