# Supplementary figures and images for: Helicobacter pylori interferes with an embryonic stem cell micro RNA cluster to block cell cycle progression
Source: Silence. 2011 Oct 25;2:7. doi: 10.1186/1758-907X-2-7 (PMC3212895; doi:10.1186/1758-907X-2-7)

Figure S1

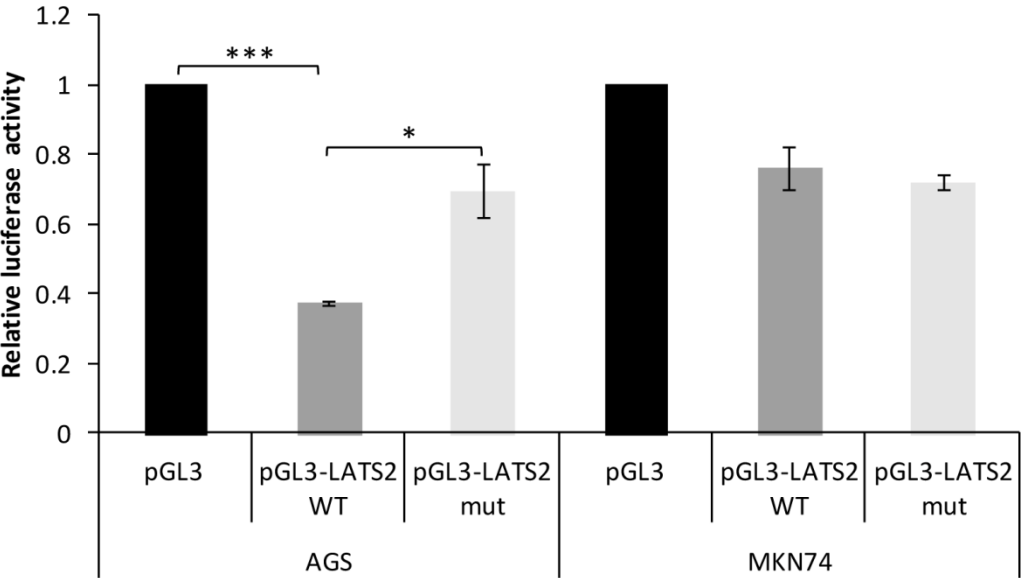

Supplement: Additional file 2 — Figure S1. Large tumor suppressor homolog 2 (LATS2) translation efficiency in AGS (high miR-372 and miR-373) and MKN-74 (low miR-372 and miR-373) cells. AGS or MKN-74 cells were plated at 0.7.105 or 105 cells/well, respectively, in a 24-well plate and transfected the following day with 100 ng/well pGL3, pGL3-LATS2 or pGL3-LATS2mut vectors [36], each mixed with 10 ng pRL-SV40 vector. Luciferase activities were measured 48 h post transfection. Bars indicate the relative firefly luciferase activity normalized to the Renilla activity and compared to pGL3. Data are mean ± SD from three independent experiments in triplicate. *P < 0.05; ***P < 0.001. [file 1758-907X-2-7-S2.PDF]

**Figure S2**

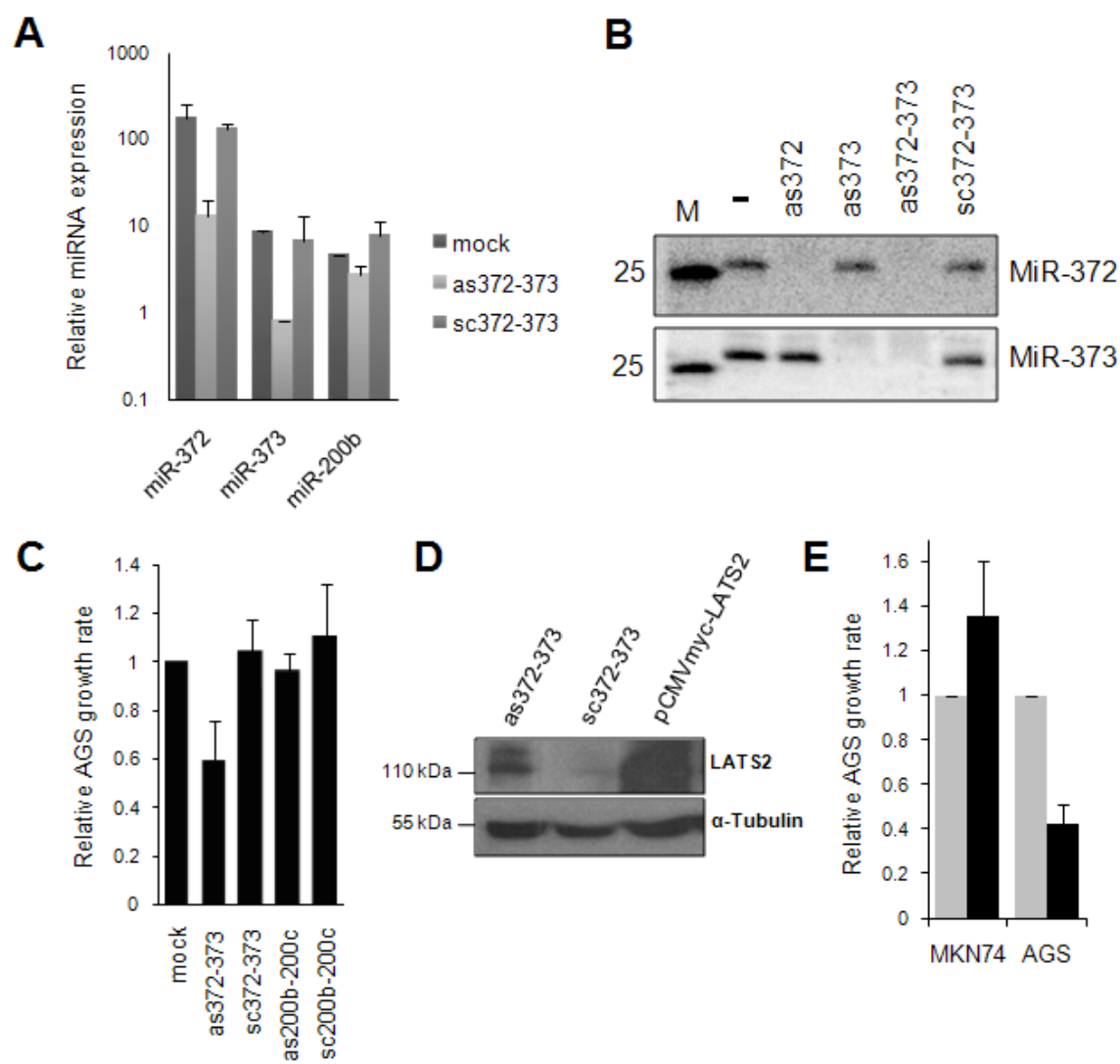

Supplement: Additional file 3 — Figure S2. Effect of antisense oligonucleotides treatment on micro RNA (miRNA) expression (A,B), large tumor suppressor homolog 2 (LATS2) expression (D) and cell growth rate (C,E). (A) Endogenous expression of miR-372, miR-373 and miR-200b was determined 48 h post transfection by quantitative real time PCR (RT-qPCR) in untreated cells (mock) or cells treated twice with 100 nM of locked nucleic acid (LNA)/DNA antisense (as372-373) or scramble (sc372-373) oligonucleotides [see Additional File 1, Table S4 for sequences]. Bars indicate relative miRNA expression normalized to U6 small nuclear RNA (snRNA) (RNU6b). Data are mean ± SD of two independents experiments. (B) Northern blot analysis of untreated cells (lane 2) or cells treated with as372 (lane 3), as373 (lane 4), the mix as372-373 (lane 5) or the mix sc372-373 (lane 6). M: DNA (bp) marker lane (Promega). (C) Comparison of AGS growth rate between non-treated (mock) or cells treated with antisense (as372-373, as200b-200c) or scrambled (sc372-373, sc200b-200c) oligonucleotides. Cells were plated at a density of 105 cells per well in a six-well plate and counted at day 2 and day 3 after plating. The results are expressed as the relative increase in cell numbers between day 2 and day 3, normalized to that of untreated AGS cells. Data are mean ± SD of three independent experiments in duplicate. (D) LATS2 immunoblot of AGS cells transfected with a pCMVmycLATS2 expression vector, or with as 372-373 or sc372-373 oligonucleotides. A total of 106 AGS cells were plated in 6 cm diameter plates and transfected the following day with 1 μg pCMVmycLATS2. Oligonucleotide treatments were performed as above. Proteic lysates were prepared 48 h post transfection and submitted to SDS-PAGE. α-Tubulin was used as a loading control. A representative immunoblot is shown. (E) Effect of ectopic expression of LATS2 on AGS or MKN74 growth rate. Black bars, cells transfected with pCMVmycLATS2; grey bars, cells transfected with pcDNA3. Cells wer [file 1758-907X-2-7-S3.PDF]

Figure S3

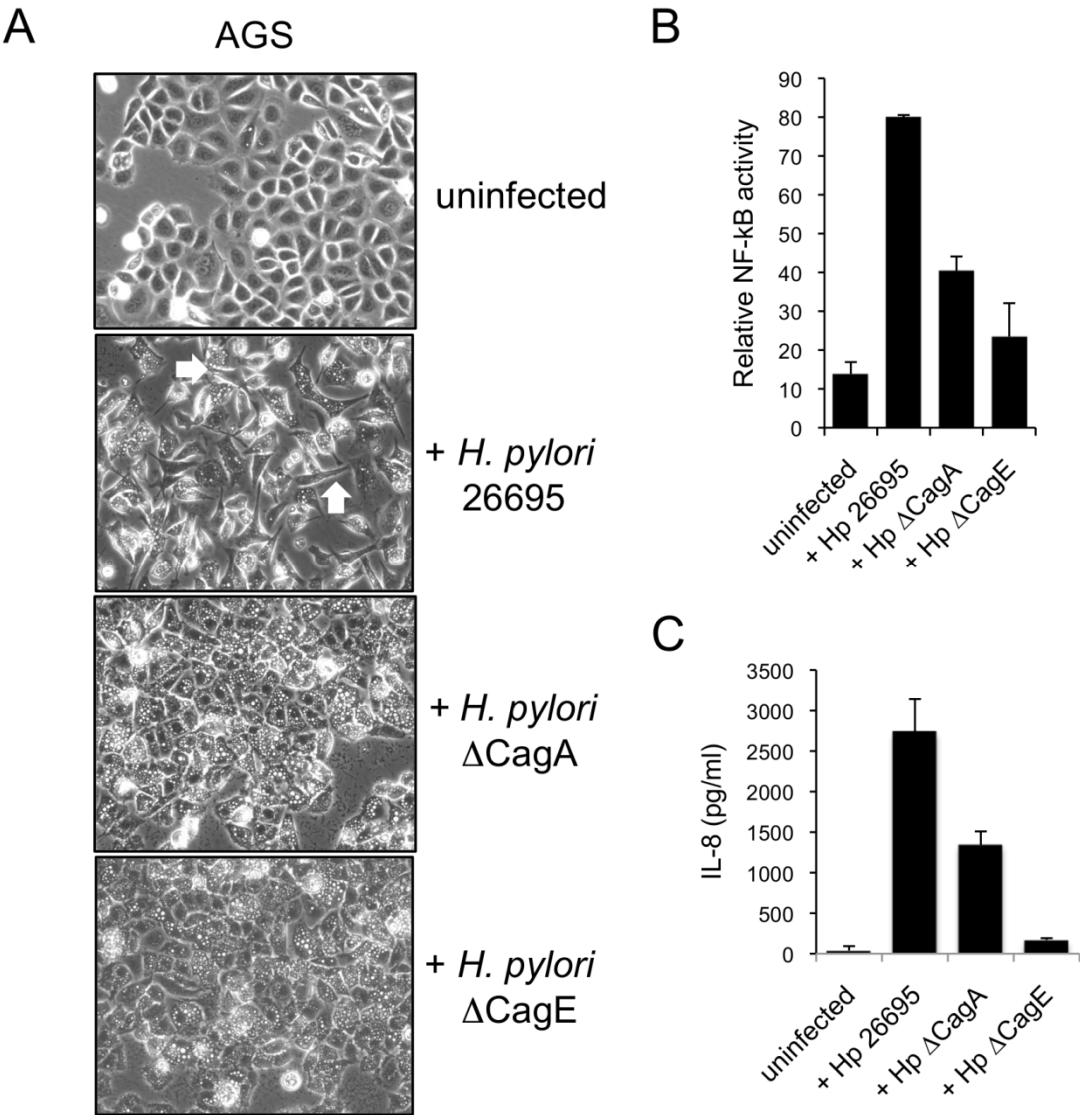

Supplement: Additional file 4 — Figure S3. Helicobacter pylori-induced morphological changes (A) and proinflammatory responses (B,C) in AGS cells. (A) Morphology of uninfected AGS cells or cells infected for 24 h with H. pylori 26695 wild-type strain or its isogenic mutants H. pylori ΔCagA and ΔCagE at multiplicity of infection (MOI) 100 was visualized by phase contrast microscopy. Uninfected AGS cells display an epithelial morphology. Coculture with H. pylori 26695 wild-type strain causes cell elongation, so called 'hummingbird' phenotype, and vacuoles. The 'hummingbird' phenotype is CagA dependent, as cells cocultured with H. pylori 26695 mutated for the CagA toxin (H. pylori ΔCagA) or unable to inject CagA (H. pylori ΔCagE) harbor only vacuoles, an effect due to the VacA toxin secreted into the culture medium. (B,C) Proinflammatory responses of AGS cells infected for 24 h with H. pylori 26695 wild-type strain or its isogenic mutants ΔCagA and ΔCagE at MOI 100 were analyzed by nuclear factor (NF)κB activation (B) and interleukin 8 (IL-8) secretion (C). As previously described [26], NFκB activation and IL-8 secretion were both CagA and Type IV Secretion System dependent. NFκB activation was monitored using the BD Mercury firefly luciferase reporters (Beckton-Dickinson) containing NFκB binding sites upstream of the firefly luciferase. Bars indicate the firefly luciferase activity normalized to Renilla luciferase, minus TAL (luciferase without promoter) activity. Data represent the mean ± SD of two independent experiments. IL-8 secreted in the culture medium was measured 24 h post infection using ELISA (R&D systems). The results represent the mean ± SD of four independent experiments. [file 1758-907X-2-7-S4.PDF]

Figure S4

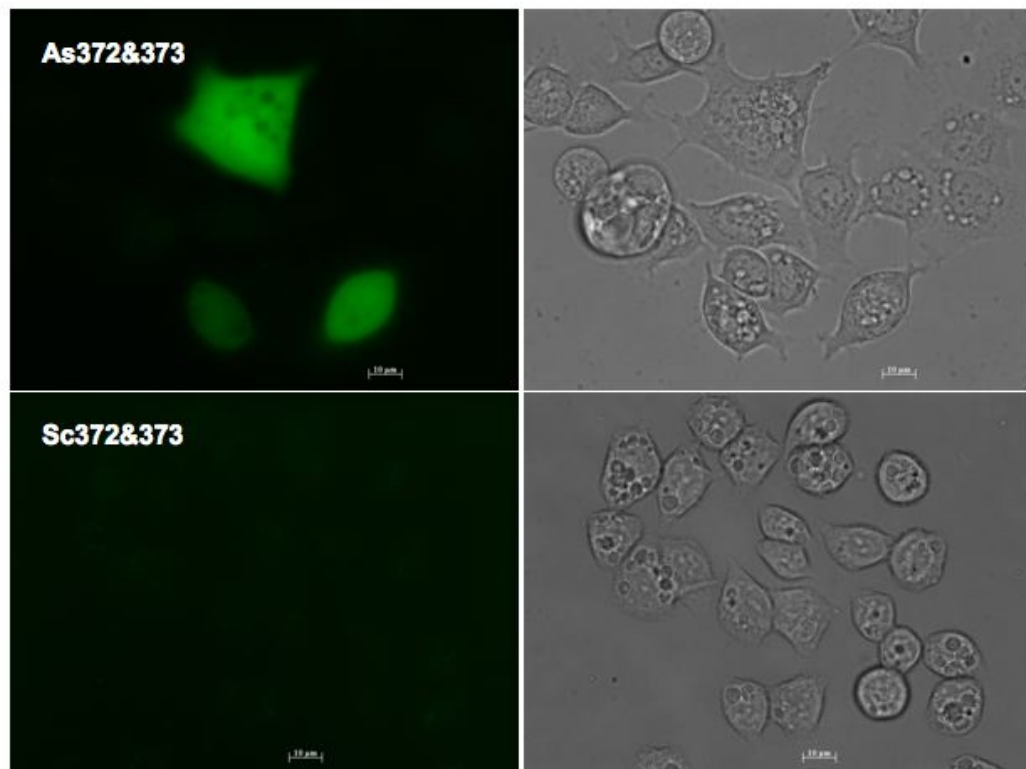

Supplement: Additional file 5 — Figure S4. Enhanced green fluorescent protein (EGFP) fluorescence of large tumor suppressor homolog 2 (LATS2) reporter-AGS cells transfected with as372-373 or sc372-373. Cells were transfected twice with 100 nM antisense or scramble oligonucleotides and observed 24 h after the last transfection on an inverted microscope (Zeiss) in phase contrast (right panels) or epifluorescence (left panels). [file 1758-907X-2-7-S5.PDF]

Figure S5

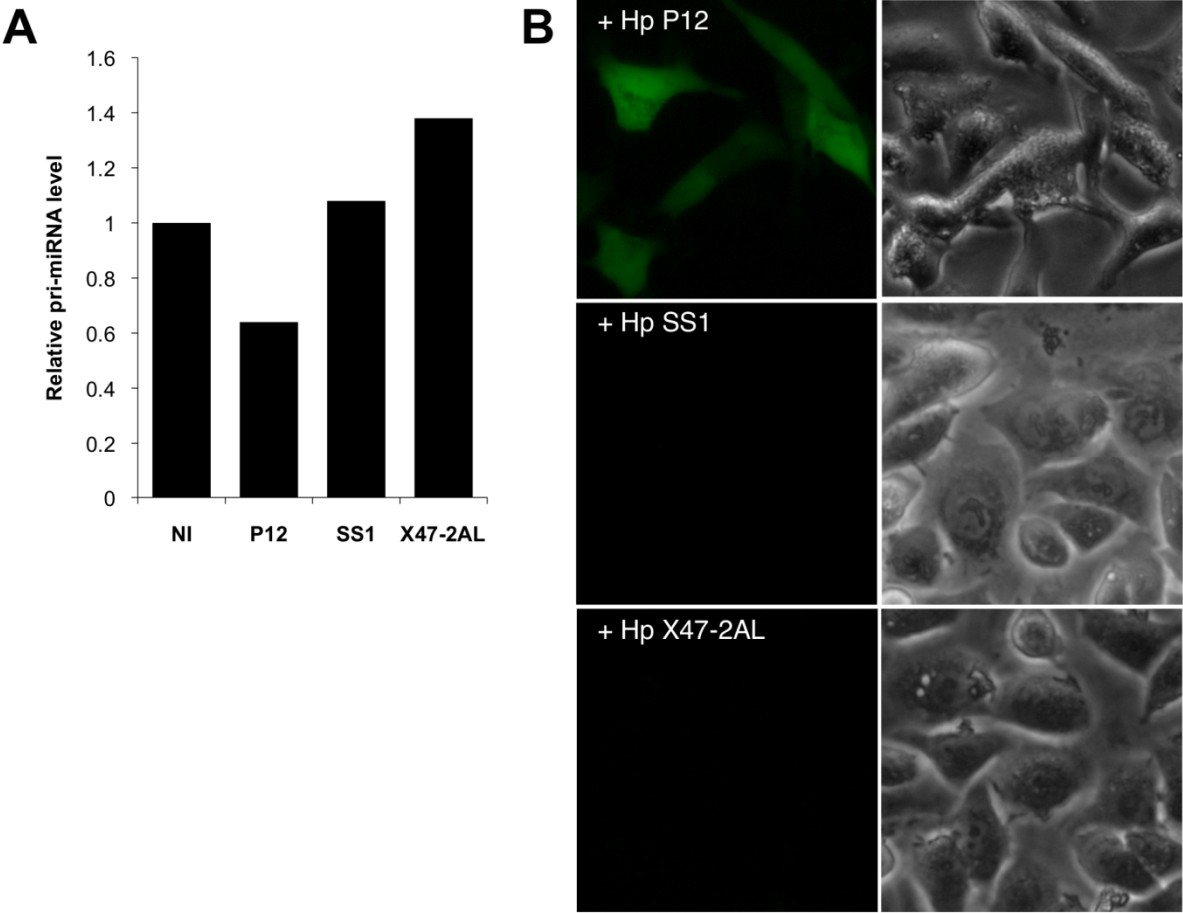

Supplement: Additional file 6 — Figure S5. Helicobacter pylori strain-specific repression of primary (pri-)miR-371-372-373 and upregulation of the enhanced green fluorescent protein (EGFP)-3' untranslated region (UTR) large tumor suppressor homolog 2 (LATS2) reporter gene. AGS cells were infected with H. pylori type I strains (P12) or type II strain (SS1, X47) at a multiplicity of infection (MOI) 100 for 24 h. (A) Expression of the pri-miR-371-372-373 was determined by quantitative real time PCR (RT-qPCR) in non-infected cells (NI) or cells infected with the different H. pylori strains. Bars indicate relative pri-micro RNA (miRNA) expression normalized to ribosomal protein P0. A representative experiment is shown. (B) EGFP fluorescence of LATS2 reporter cells infected or not with H. pylori strains. Cells were observed 24 h post infection on an inverted microscope (Zeiss) in phase contrast (right panels) or epifluorescence (left panels). [file 1758-907X-2-7-S6.PDF]

Figure S6

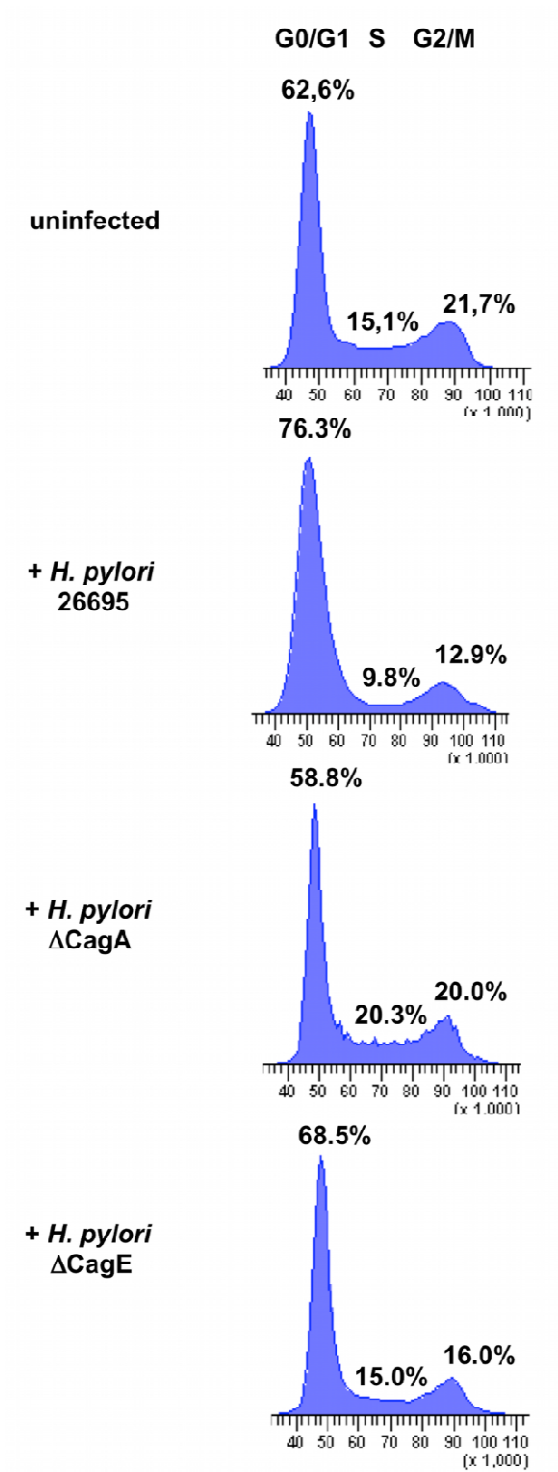

Supplement: Additional file 7 — Figure S6. CagA-dependent accumulation of Helicobacter pylori-infected AGS cells in G0/G1 phase. Cellular DNA contents of uninfected AGS cells or cells infected for 24 h with wild-type, ΔCagA or ΔCagE H. pylori were stained by iodide propidium and analyzed by flow cytometry. [file 1758-907X-2-7-S7.PDF]
